# Longitudinal Bottom-Up Proteomics of Serum, Serum Extracellular Vesicles, and Cerebrospinal Fluid Reveals Candidate Biomarkers for Early Detection of Glioblastoma in a Murine Model

**DOI:** 10.3390/molecules26195992

**Published:** 2021-10-02

**Authors:** Francesco Greco, Federica Anastasi, Luca Fidia Pardini, Marialaura Dilillo, Eleonora Vannini, Laura Baroncelli, Matteo Caleo, Liam A. McDonnell

**Affiliations:** 1 Institute of Life Sciences, Sant’Anna School of Advanced Studies, 56127 Pisa, Italy; fr.greco@santannapisa.it; 2 Fondazione Pisana per la Scienza ONLUS, 56017 San Giuliano Terme, Italy; federica.anastasi@sns.it (F.A.); lf.pardini@fpscience.it (L.F.P.); mari.dilillo@gmail.com (M.D.); 3 NEST Laboratories, Scuola Normale Superiore, 56127 Pisa, Italy; 4 Department of Chemistry and Industrial Chemistry, University of Pisa, 56124 Pisa, Italy; 5 CNR, Neuroscience Institute, 56124 Pisa, Italy; e.vannini@in.cnr.it (E.V.); baroncelli@in.cnr.it (L.B.); caleo@in.cnr.it (M.C.); 6 Fondazione Umberto Veronesi, 20122 Milano, Italy; 7 IRCCS Fondazione Stella Maris, 56018 Calambrone, Italy; 8 Dipartimento di Scienze Biomediche, Università di Padova, 35131 Padova, Italy

**Keywords:** glioblastoma, biomarkers, proteomics, longitudinal, serum, extracellular vesicles, CSF

## Abstract

Glioblastoma Multiforme (GBM) is a brain tumor with a poor prognosis and low survival rates. GBM is diagnosed at an advanced stage, so little information is available on the early stage of the disease and few improvements have been made for earlier diagnosis. Longitudinal murine models are a promising platform for biomarker discovery as they allow access to the early stages of the disease. Nevertheless, their use in proteomics has been limited owing to the low sample amount that can be collected at each longitudinal time point. Here we used optimized microproteomics workflows to investigate longitudinal changes in the protein profile of serum, serum small extracellular vesicles (sEVs), and cerebrospinal fluid (CSF) in a GBM murine model. Baseline, pre-symptomatic, and symptomatic tumor stages were determined using non-invasive motor tests. Forty-four proteins displayed significant differences in signal intensities during GBM progression. Dysregulated proteins are involved in cell motility, cell growth, and angiogenesis. Most of the dysregulated proteins already exhibited a difference from baseline at the pre-symptomatic stage of the disease, suggesting that early effects of GBM might be detectable before symptom onset.

## 1. Introduction

Glioblastoma Multiforme (GBM, World Health Organization (WHO) grade IV astrocytoma [1]) is the most malignant glial tumor and is associated with a very poor prognosis, with a median survival of just 15 months [2]. The vast majority of GBM occurs de-novo in older patients (primary GBM), with only 5% arising from low-grade astrocytoma in younger patients (secondary GBM) [3]. Current GBM treatments are limited to complete tumor resection followed by radiotherapy and adjuvant chemotherapy [3].

GBM diagnosis relies on neuroimaging techniques, often performed after the clinical presentation of symptoms and thus showing a considerable size and differentiation of the mass [4,5]. Extended resections have been shown to increase the median survival time, but can also cause motor deficits and functional decline [4].

It is widely established that tumor stage and grade determine patient outcomes and patient treatment [5,6] in many types of tumors. For instance, earlier diagnosis of breast cancer allows the tumor to be treated in the initial stage, significantly improving patient prognosis [7]; the introduction of screening for the prostate-specific antigen has decreased prostate cancer lethality by 21% [8,9]; and screening for α-fetoprotein [10,11] has enabled earlier detection and concomitant improvements in the outcome of hepatocellular carcinoma [12,13]. A lack of biomarkers for earlier-stage GBM has impeded any investigation of possible beneficial effects of early diagnosis in terms of treatment outcome.

Biomarker discovery for patient diagnosis has been the subject of a wide body of research, utilizing many diverse omics technologies and patient materials [14,15,16,17,18]. To be practical, an assay for early diagnosis should be based on accessible body fluids, such as urine, saliva, serum/plasma, and cerebrospinal fluid (CSF), or performed in material derived from biological fluids such as extracellular vesicles (EVs) and circulating tumor cells [19].

The identification of biomarkers for earlier detection of GBM is hampered by the absence of early-stage patient material because patients are diagnosed when the disease is already advanced. Animal models likely represent the only solution to investigate early-stage GBM because they provide the possibility to perform longitudinal analyses, including at pre-symptomatic stages.

Murine models are well established for GBM [20]; syngenic GBM models, in contrast to human transplant models, do not require an immune-deficient system and can mimic the interaction between GBM and the brain tissue environment [21]. Despite its advantages, the longitudinal analysis of biofluids in rodent models is challenging owing to the limited sample volume that may be obtained at each time point. The amount of biofluid that may be withdrawn is determined by the number of time points, the weight of the animal, and the recovery time between collections, to preserve animal welfare and reduce sampling invasiveness [22]; for instance, just 75 µL of serum may be obtained every 14 days for adult mice [23]. Highly sensitive approaches for the molecular characterization of the samples obtained from each time point, from each individual animal, are needed for this type of analysis.

Here, a set of microproteomics procedures are reported for the longitudinal proteomics analysis of serum, small extracellular vesicles isolated from serum (serum-sEV), and cerebrospinal fluid. The workflows were applied for the investigation of early-stage biomarkers in a recently developed GBM mouse model, in which glioma-induced early dysfunction is longitudinally monitored via behavioral analyses of motor function.

## 2. Results

The frontal cortex is the most common anatomical location of glioma [24]. GBM induction in the primary motor cortex (located in the frontal lobe) induces motor deficits and neurological symptoms in mice [25]. Motor function was monitored through behavioral tasks to chart tumor progression, and thereby define pre-symptomatic and symptomatic stages.

**Figure 1:**
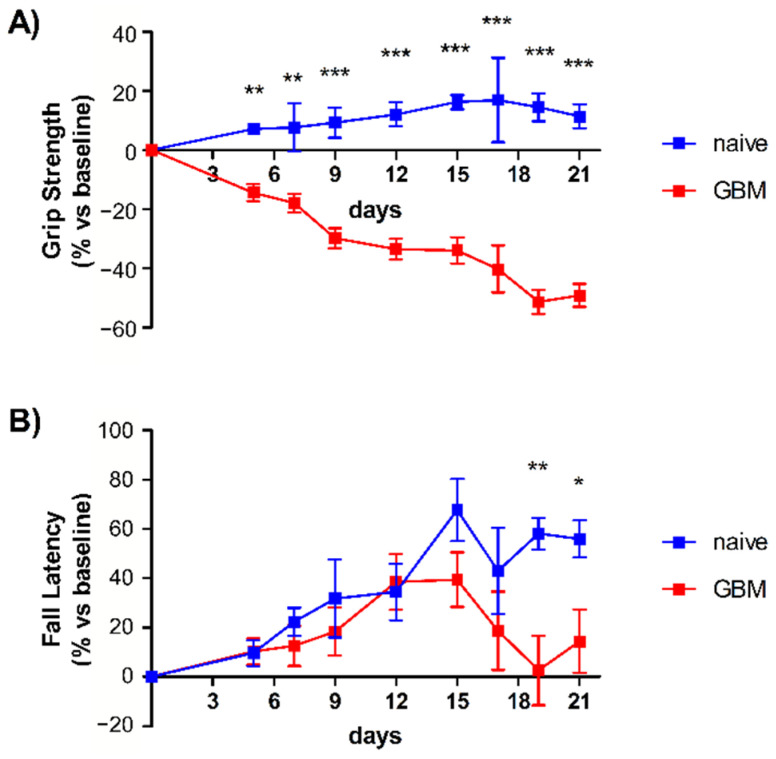
Motor tests and assessment of tumor progression. (**A**) Grip strength test. GBM mice (red line, *n* = 6) displayed a significant deterioration of grip strength starting from day 5 with respect to naïve mice (blue line, *n* = 9), which instead showed a slight improvement in task performance over the time course of the study and in accordance with data previously reported. (**B**) Rotarod test. Both groups showed an increase in their motor performance until day 12 due to the learning component of this motor task [25]. However, from day 15, after tumor induction, the GBM mice (red line, *n* = 6) exhibited worse performance, indicative of significant motor dysfunction with respect to naïve animals. Data are expressed as mean ± SEM. * *p* < 0.05, ** *p* < 0.01, *** *p* < 0.001 using a two-way repeated-measures ANOVA with Holm–Sidak post hoc correction.

The results from Grip strength and Rotarod tests are shown in Figure 1 and were consistent with previous findings, namely a decrease in grip strength and motor performance with tumor progression [25]. The pre-symptomatic stage of GBM was defined as 12 days post inoculation, when the onset of motor symptoms had begun but was not completely manifest. Blood and CSF withdrawal was performed 15 days prior to inoculation (baseline), and 12 days and 21 days afterward (T1 and T2, respectively). The tumor volume at the pre-symptomatic and symptomatic stages of tumor progression for the syngeneic mouse model used in this study was previously evaluated [25] and confirmed that tumor growth mainly occurs between 14 and 23 days post-inoculation. Figure S1 shows the Hoechst-stained brain section of a glioma-bearing mouse 23 days after tumor implantation (coronal brain section, 45 μm, 1:500 *v*/*v*).

### 2.1. Serum, Serum sEVs, and CSF Proteomics

Longitudinal analysis of circulating proteins from small animal models is challenging due to the limited amount of biofluids that can be sampled per time point. The amount of biofluid that may be withdrawn is determined by the number of time points, the weight of the animal, and the recovery time between collections, to preserve animal welfare and reduce sampling invasiveness [22]. The analytical methods were first optimized in order to process the low amounts of biofluids that could be obtained from each animal at each time point.

In-depth proteome coverage of serum must contend with the high dynamic range of protein concentration [26,27]. An SDC-based proteomics workflow that includes immunodepletion of highly abundant proteins and sample fractionation after TMT labeling was developed. Using this protocol, we were able to quantify more than 600 serum proteins from just 15 µL of serum.

The de-facto gold standard for EV isolation, ultracentrifugation [28], is prone to vesicle aggregation and disruption. The typical sample volume for ultracentrifugation is several milliliters [29,30], far more than the quantity available in longitudinal studies involving small animals [31]. For these reasons, we used an SEC-based protocol that was able to efficiently isolate sEVs from just 50 µL of serum, thereby enabling longitudinal analysis of mice [32]. An average of 1.8 µg of sEV of proteins could be isolated per time point. These sEV proteins were then processed for proteomics analysis using a modified SP3 protocol [33], which led to the identification of 274 protein groups. The combination of these protocols enabled the parallel analysis of serum and serum sEVs from each time point of each animal (serum amount available ≤ 75 μL [22]). The serum sEV data included 99 proteins that were not present in the serum dataset (Figure S2A and Supplementary Material 4). Furthermore, a comparison of the sEV dataset with the ExoCarta Top 100 database of exosome markers revealed the presence of 26 exosome markers, 9 of which were identified in all samples (Supplementary Material 4).

The same SP3 workflow was adapted for the analysis of the longitudinal CSF samples, for which only around 5 µL were available per time point and corresponded to less than 2 µg of protein (see methods section for more details). The quality of CSF collection was assessed by comparing the proteins identified in all CSF samples with proteins previously reported in the CSF of murine models of stroke and neurodegenerative diseases [34,35,36]. The majority of proteins identified in the CSF dataset (circa 93%) were in agreement with at least one of the reported datasets (Figure S2B and Supplementary Material 4), supporting the quality of CSF collection, processing, and analysis.

Despite the low amount of serum, immunodepletion of high-abundance proteins and high-pH fractionation allowed the identification of 644 protein groups. This result is comparable with other studies involving higher serum amounts and more extensive immunodepletion and fractionation [37,38] using similar instrumentation. The optimized SP3 workflow allowed the quantification of 274 serum sEV proteins. CSF samples were compared to a high-pH fractionated pooled sample using the match-between-runs function of MaxQuant. This approach led to the identification of 3002 protein groups, consistent with previous studies in which extensive fractionation was used to increase proteome coverage [39,40].

### 2.2. Longitudinal Proteomics Analysis of Mouse Serum, Serum sEVs and CSF

**Figure 2:**
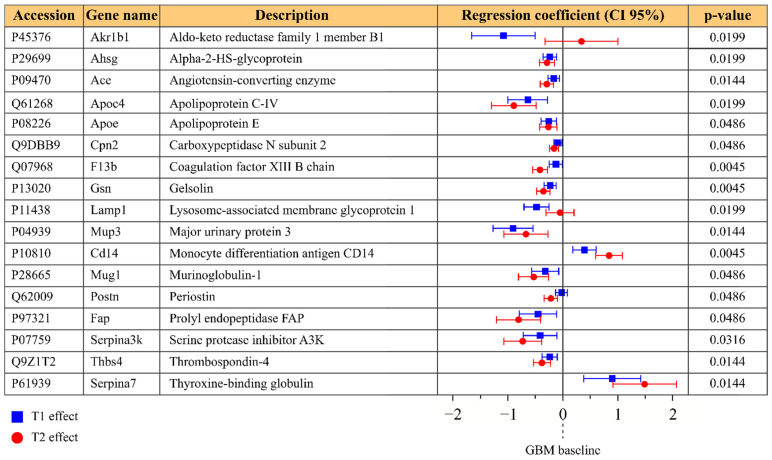
Protein groups that exhibited significant differences in the serum dataset; *p*-values refer to the H0 hypothesis that all coefficients of the model are zero. Regression coefficients are reported in blue for a T1 (pre-symptomatic) effect and in red for a T2 (symptomatic) effect, with their confidence interval at 95%. Regression coefficients represent the fold change compared to the baseline.

The longitudinal proteomics dataset from serum consisted of 18 points, corresponding to the baseline, T1, and T2 time points measured from six individual glioma-bearing mice. The LME model was individually fitted to each protein, under the condition that at least four valid values were present for each time point. The model was fitted to the log2 transformed normalized intensities of 612 protein groups; of these, 38 were discarded because either the model residuals or the random effects were not normally distributed, and 281 were discarded due to insufficient valid values. Figure 2 shows 17 protein groups that exhibited a significant change in level during GBM progression, with respect to the baseline. In most cases, the fold change at the pre-symptomatic stage (T1) was less than that at the symptomatic stage (T2).

**Figure 3:**
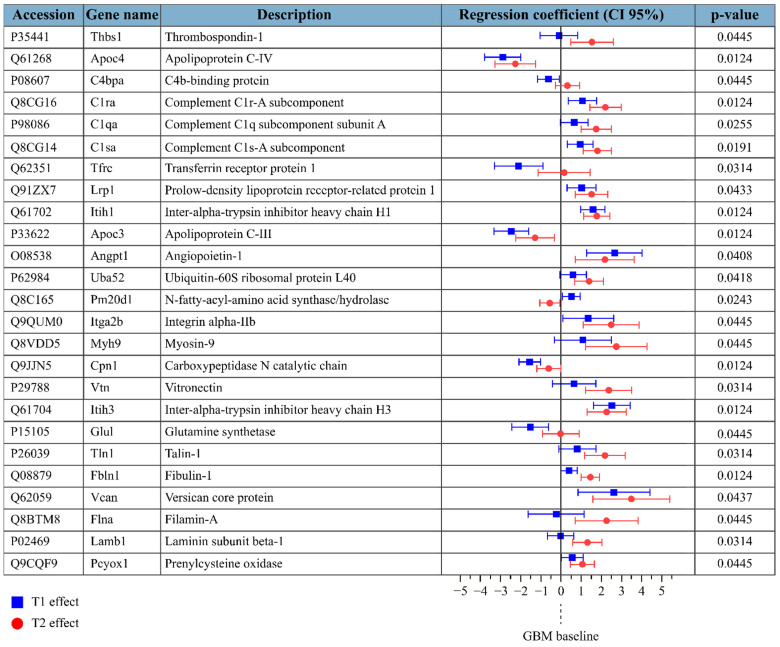
Protein groups that exhibited significant differences in the serum sEV dataset; *p*-values refer to the H0 hypothesis that all coefficients of the model are zero. Regression coefficients are reported in blue for a T1 (pre-symptomatic) effect and in red for a T2 (symptomatic) effect, with their confidence interval at 95%. Regression coefficients represent the fold change compared to the baseline.

For the sEV dataset, time points were complete for all mice, except for a missing T2 and a missing baseline (from different mice). Protein intensities were log2 transformed and normalized by median subtraction. The LME model was fitted only if at least three baselines, three T1, and three T2 had valid values for the protein. The serum sEV dataset included quantitative information for 211 protein groups, 13 of which were then omitted because the residuals or random effects were not normally distributed and 102 due to insufficient valid values. Figure 3 shows 25 protein groups that exhibited a significant change in level during GBM progression. Again, the fold change at the pre-symptomatic stage (T1) was less than that at the symptomatic stage (T2) for the majority of proteins. Only one protein, apolipoprotein C-IV, exhibited significant differences in levels in both the serum and serum sEV datasets; different subunits of carboxypeptidase N were found to be differentially detected in GBM in the longitudinal serum dataset (carboxypeptidase subunit 2) and the longitudinal serum sEV dataset (carboxypeptidase catalytic chain).

**Figure 4:**
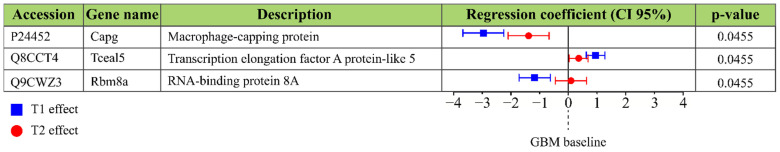
Protein groups that exhibited significant differences in the CSF dataset; *p*-values refer to the H0 hypothesis that all coefficients of the model are zero. Regression coefficients are reported in blue for a T1 (pre-symptomatic) effect and in red for a T2 (symptomatic) effect, with their confidence interval at 95%. Regression coefficients represent the fold change compared to the baseline.

Five mice were used to estimate time point effects for CSF. Three mice missed the T2 point, while one mouse missed both baseline and T1, so the model was fitted only for proteins with valid values for all samples. Protein intensities were log2 transformed and normalized by median subtraction. We identified 3002 protein groups, of which 2081 lacked sufficient values and were discarded. 74 protein groups were discarded due to the non-normal distribution of either residuals or random effects. Figure 4 shows 3 protein groups that exhibited a significant change in level during GBM progression, with respect to the control animals. Again, the fold change at the pre-symptomatic stage (T1) was less than that at the symptomatic stage (T2).

Despite the very low volumes available from the longitudinal sampling of biofluids from individual animals of a murine GBM model, the longitudinal analysis of serum, serum sEVs, and CSF revealed 44 protein groups that exhibited a significant temporal change during GBM progression (Figure 2, Figure 3 and Figure 4). In particular, 22 proteins decreased and 21 proteins increased in their concentration in at least one of the disease stages (T1 or T2) with respect to the baseline. Only one protein (*N*-fatty-acyl-amino acid synthase/hydrolase) increased its fold change at T1 but showed a significant decrease at T2 compared to baseline.

**Figure 5:**
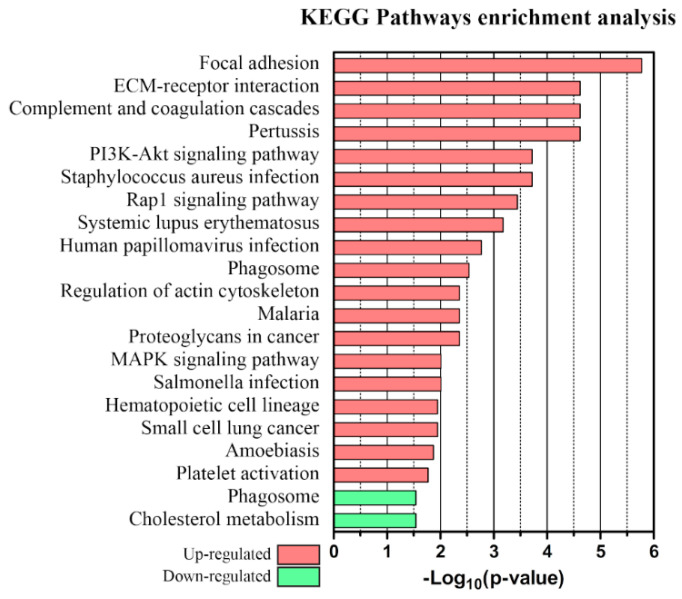
KEGG pathways GO enrichment analysis of proteins upregulated (red) or downregulated (green) in at least one time point.

Upregulated and downregulated proteins were each subjected to an enrichment analysis using STRING v.11. Up- and down-regulated pathways of proteins dysregulated in at least one time point are shown in Figure 5 along with the corresponding *p*-values. Up- and down-regulated pathways of proteins dysregulated at the presymptomatic stage are shown in Figure S3.

## 3. Discussion

The discovery of circulating biomarkers for earlier diagnosis of GBM is limited by the lack of availability of body fluids and tissues from patients prior to diagnosis. In this context, a longitudinal study on small animal models enables the identification of candidate biomarkers, despite facing difficulties linked to the low sample amount involved in the analysis.

Here the sample preparations for serum, serum sEV, and CSF proteomics were scaled down to enable the longitudinal analysis of individual animals. The 15 µL serum used for the serum analysis and the 50 µL for the serum sEV analysis allowed the longitudinal analysis of serum and serum sEVs of the same individual animals at the same time points. Mouse CSF proteomics is usually limited by the low sample amount [36] and by the presence of CSF high-abundance proteins [41]; in the case of longitudinal studies, the sample amount is reduced even further because of issues related to sampling invasiveness. In this study, the SP3 microproteomics workflow was adapted to CSF samples, enabling the analysis of three time points across 36 days.

Experimental difficulties associated with repeated samplings of small animal models means missing time points are not uncommon, particularly for CSF in which scarring can make sampling at later time points difficult or lead to blood contamination. Furthermore, the low volume of biofluid available at each time point from each animal [22] means the quantitative proteomics data can be prone to missing values because of the stochastic nature of data-dependent acquisition. Here, the match-between-runs method was used to reduce the severity of this effect for the serum sEV and CSF datasets, but data-independent methods could help further reduce missing values [42,43]. Nevertheless, such longitudinal analyses must contend with missing values; the linear mixed effects model used here was chosen because it models longitudinal changes in expression levels, incorporates statistical checks on the distribution of residuals and effects, and is robust to missing values [44]. LME has previously been used to investigate temporal changes in protein expression levels associated with clinical decline and childhood development [45,46].

When the LME method was applied to serum, serum sEVs, and CSF datasets, it led to the identification of more than forty dysregulated proteins. It is interesting to note that significant proteins in the three datasets exhibit little overlap, with just a single significant protein in common between serum and sEVs despite the serum origin of the vesicles. When analyzing whole serum, the protein signals from serum sEVs would be masked by the much more abundant non-sEV serum proteins (which is the reason we implemented a separate sEV purification strategy to target serum sEV proteins). SEC isolation and microproteomics are needed to explore the protein content of sEVs without the interference of more abundant serum proteins. The unbiased analysis of sEVs is particularly relevant here, since vesicles are a promising source of GBM markers [47,48]. Among the proposed sEVs biomarkers, we identified several proteins that have previously been reported as derived from GBM exosomes. Six out of nineteen upregulated sEVs proteins were found in exosomes derived from two different human GBM cell lines [47] (Tln1, Myh9, Thbs1, Flna, Vcan, and Lamb1), and five markers that the authors associate with GBM invasiveness were present in the sEVs dataset but were not dysregulated (App, Ecm1, Gapdh, Itgb1, and Mvp). Six of the upregulated sEVs markers were detected in the exosomes of four out of five GBM cell lines in another proteomics study [48] (Tln1, Myh9, Thbs1, Flna, Fbln1, and Vtn). Several of the proposed markers from sEVs are commonly found in GBM-derived exosomes. The enrichment analysis of proteins dysregulated in at least one time point showed a significant increase in cancer-related pathways, involving cell motility and invasion, proliferation, and angiogenesis. The circulating biomarkers identified here can be attributed to leakage from the tumor mass through the damaged blood–brain barrier [49,50] but also to the effects of the tumor mass on the surrounding environment.

Enrichment analysis of upregulated proteins revealed increased expression of proteins involved in focal adhesion complexes, ECM–receptor interactions, and proteoglycans in cancer pathways (focal adhesion: FDR 1.68e-06, Thbs1, Vtn, Itga2b, Lamb1, Flna, and Tln1; ECM-receptor interaction: FDR 2.41e-05, Thbs1, Vtn, Itga2b, and Lamb1; proteoglycans in cancer: FDR 0.0044, Vtn, Thbs1, and Flna). Among the proteins involved in the interaction with the ECM were structural components (FDR: 3.9e-04, Lamb1, Fbln1, and Vcan), membrane proteins (integrin αIIb), and cytoskeletal proteins (Tln1, Flna, and Myh9). The glycoprotein vitronectin is involved in cell adhesion, growth, and migration [51] and has been shown to protect GBM cells from apoptosis [52] and to promote glioma cell migration [53,54]. An increase in circulating vitronectin has previously been proposed as a diagnostic and prognostic biomarker for glioma [55]. Filamin-A is a cytoskeleton protein involved in actin binding and plays a crucial role in interacting with integrins during cell migration [56]. Filamin-A has also been shown to be involved in GBM cell invasion and motility [57] and has been previously reported as overexpressed in the plasma of GBM patients [56]. Here, the proteins vitronectin, filamin-A, thrombospondin-1, talin-1, and laminin subunit beta 1 were found at increased levels in the serum sEVs of GBM, particularly at the symptomatic stage (T2).

Upregulated proteins were involved in PI3K-Akt, MAPK, and Rap1 pathways (PI3K-Akt, FDR 1.9e-04, Vtn, Itga2b, Angpt1, Thbs1, Lamb1; MAPK, FDR 0.0098, Flna, Angpt1, Cd14, Rap1: FDR 3.6e-04, Thbsp1, Angpt1, Itga2b, Tln1). The PI3K-Akt pathway is one of the most frequently dysregulated pathways in cancer, involved in cell growth, proliferation, apoptosis inhibition, and angiogenesis [58,59]. Of these proteins, angiopoietin-1 is involved in vessel stabilization [58] and modulating aberrant vessel development in GBM [59], while thrombospondin-1 has a role in angiogenesis [60] and is overexpressed in high-grade gliomas, where it modulates expansion and invasion [61]. The MAPK pathway is linked to immune and stress responses [62] while the Rap1 pathway is involved in cell–cell and cell–ECM interactions [63]. The monocyte differentiation antigen CD14, one of the proteins contributing to the over-representation of the MAPK pathway, has been reported to increase with grade in astrocytomas [64]. Both angiopoietin-1 in serum sEVs and the monocyte differentiation antigen CD14 in serum increased their levels at the pre-symptomatic stage of GBM (T1) and at the later symptomatic stage (T2); thrombospondin 1 was only detected at increased levels in serum sEVs at the symptomatic stage.

The complement and coagulation cascade (FDR: 2.41e-05, Vtn, C1qa, C1ra, and C1s1) is over-represented in the upregulated proteins. C1qa, C1ra, and C1s1 are involved in the classical pathway of complement activation [65]. The classical pathway is inhibited by C4b binding protein and by carboxypeptidase N [66]. C4b binding protein was detected at reduced levels in serum sEVs at the pre-symptomatic stage; the concentration of both carboxypeptidase N subunits was reduced at the pre-symptomatic and symptomatic stages. Complement activation in the context of chronic inflammation can promote tumor progression and metastasis [67], and serum levels of C1q have been reported to be increased in GBM patients [68].

Enrichment analysis of downregulated proteins revealed an over-representation in biological processes of regulation of tissue remodeling (Ahsg, Tfrc, Thbs4, and FDR 6.7 × 10^−3^), tissue regeneration (Gsn, Postn, and FDR 0.0279), and wound healing (F13b, Gsn, Postn, and FDR 0.0418); results are included in Supplementary Material 3. Alpha-2-HS-glycoprotein (Ahsg or fetuin) was found to be downregulated in serum at both the pre-symptomatic and symptomatic stages; low serum alpha-2-HS-glycoprotein levels have been reported as indicative of a shorter survival time in GBM patients [69]. Gelsolin (Gsn) was also decreased in serum at the pre-symptomatic and symptomatic stages, and decreased circulating gelsolin levels have also been reported in GBM patients [69,70] while its tissue expression decreased with grade in astrocytomas [71]. Among the other proteins involved in tissue remodeling, we found prolyl endopeptidase FAP (Fap) decreased at both pre-symptomatic and symptomatic stages in the serum dataset. Prolyl endopeptidase FAP reduces scar resolution, activating plasmin inhibitors and blocking fibrinolysis, though questions remain regarding its role in cancer [72].

When the same enrichment analysis was performed using only those proteins exhibiting significant differences at the pre-symptomatic stage, the number of differentially regulated pathways was smaller (Figure S3), likely due to the smaller number of proteins used in the analysis, because some of the proteins contributing to cancer-related pathways like Vtn, Thbs1, Flna, Lamb1, and Tln1 did not exhibit significant differences at the pre-symptomatic stage. Nevertheless, PI3K-Akt and Rap1 signaling pathways were significantly upregulated even at the pre-symptomatic stage. The observation that some proteins like Akr1b1, Lamp1, Tfrc, and Glu1 were found to increase their concentration at the pre-symptomatic stage of the disease but returned to baseline at the more advanced stage is intriguing but not fully understood at this time, owing to the many factors and cell types involved in tumor growth and the non-trivial link between the protein profile of circulating fluids and tumor stage.

Some of the dysregulated proteins detected in this study have previously been reported as candidate diagnostic markers of advanced-stage GBM. Our study suggests that these proteins could be diagnostic at earlier stages of the disease, opening the possibility of large screening tests in at-risk populations. GBM is usually diagnosed at an advanced stage and thus little is known about the effects of early treatment that could tackle cancer before the establishment of high tumor heterogeneity [73,74]. The early detection of GBM, together with appropriate therapies, might have a beneficial effect on the median survival of GBM patients.

### Study Strength and Limitations

The discovery of circulating biomarkers for earlier diagnosis of GBM is limited by the unavailability of body fluids and tissues from patients before the onset of unequivocal symptoms and patient diagnosis. In this context, a longitudinal study on small animal models is much more efficacious and significantly less costly. Moreover, longitudinal analysis allows one to extract the maximum information from each animal, fulfilling the replace, reduce, and refine (3Rs) animal welfare requirements. One of the strengths of the study is the scaling down of serum, sEV, and CSF proteomics analysis, to adapt the sample volumes to longitudinal studies on small animals in which individual animals are individually analyzed.

Extracellular vesicles play an important role in cell-to-cell communication and cancer development and are a promising source of biomarkers [75,76,77]. EVs carry cell-specific information and preserve their cargo during circulation [76]. For this reason, we sought to compare the information obtained from serum and serum sEV from the same sample. Interestingly, the majority of biologically relevant GBM-related proteins were found in sEVs, suggesting that circulating vesicles are a promising source of biomarkers for early-stage GBM.

Mouse CSF proteomics is usually limited by the low sample amount [36]; in the case of longitudinal studies, the sample amount is reduced further because of issues related to sampling invasiveness. In this study, the SP3 microproteomics workflow was adapted to CSF samples, enabling the analysis of three time points across 36 days.

Another strength of the present study is that tumor inoculation in the primary motor cortex enabled non-invasive monitoring of tumor development using motor tests [25]. Sampling timing was thus chosen based on physical evidence of GBM’s early onset and progression.

The GBM model was chosen because it enabled the non-invasive monitoring of tumor progression. During the development of the model, it was demonstrated that effects due to the surgical procedure had passed by day 9 [25], significantly earlier than the pre-symptomatic stage used here, day 12. Nevertheless, the inclusion of sham animals, in which the entire procedure is repeated using the inoculation medium but with no GBM cells, would better control for molecular effects due to the surgical procedure but would require more animals.

The small number of animals used here will have limited the statistical power of the analysis, and thereby underestimated the biomolecular changes associated with tumor progression. Nevertheless, many proteins that were dysregulated in this study have previously been reported as biomarkers of advanced GBM or are associated with GBM [55,56,69,70].

## 4. Materials and Methods

### 4.1. Materials

LC-MS-grade water was purchased from VWR International (Radnor, PA, USA). Reagents for TMT10plex, the MicroBCA protein assay, and Pierce Concentrator 3KDa MWCO 0.5 mL were purchased from Thermo Fisher Scientific (Rockford, IL, USA). The protease inhibitors cocktail cOmplete^TM^ Mini EDTA-free EASYpack was acquired from Roche (Basel, Switzerland). Lysyl endopeptidase C (Lys-C), mass spectrometry grade, was bought from Wako (Neuss, Germany).

Furthermore, the 0.22 µm spin filters, AssayMap BRAVO 5 µL cartridges (C18 and RPS), and immunodepletion Multiple Affinity Removal Spin Cartridge MOUSE-3 were purchased from Agilent Technologies (Santa Clara, CA, USA).

Vivaspin 500 3 kDa MWCO filters were bought from Sartorius (Gottingen, Germany). Trypsin/Lys-C mix Mass Spec grade was purchased from Promega (Madison, WI, USA), and size Exclusion Chromatography columns (qEV-70 nm) were obtained from IZON (Christchurch, NZ, USA).

All other reagents were purchased from Sigma-Aldrich (Saint Louis, MO, USA).

### 4.2. Animal Model

#### 4.2.1. Experimental Design

**Figure 6:**
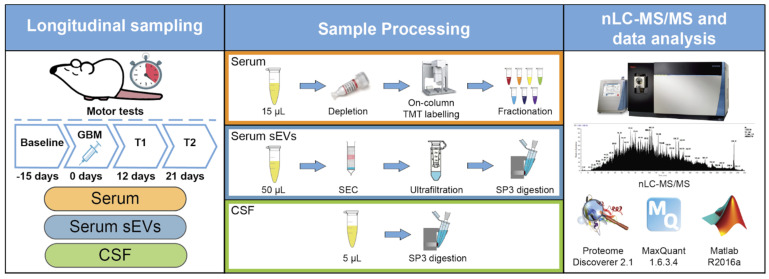
Summary of the experimental workflow. A GBM syngenic murine model was used in the study and tumor progression was monitored by motor tests. Serum, serum sEVs, and CSF samples were collected for baseline, pre-symptomatic (T1), and late-stage (T2) GBM progression. Proteins were extracted, processed, and analyzed by nLC-MS/MS. Proteins were identified using Proteome Discoverer 2.1 and MaxQuant 1.6.3.4. A linear mixed effect model for longitudinal data analysis was implemented in Matlab v.R2016a.

Figure 6 shows a summary of the experimental workflow followed here. The experiments are based on an established GBM model [25] in which tumor inoculation in the motor cortex enables non-invasive monitoring of tumor progression by motor tests. Deficits in the grip strength and rotarod tests were used to define pre-symptomatic and symptomatic stages of the disease.

#### 4.2.2. Tumor Induction

Adult (age > postnatal day 60) C57BL/6J mice were used for this study. Six mice were used for serum and sEVs analysis (three males and three females) while five mice were used for CSF analysis (three males and two females). The animals were bred at the CNR Neuroscience Institute (Pisa) animal facility under 12 h light/dark cycles, with ad libitum availability of food and water. All experiments were performed in compliance with the EU Council Directive 2010/63/EU on the protection of animals used for scientific purposes and were approved by the Italian Ministry of Health (authorization number 260/2016-PR, 11 March 2016). The murine glioma GL261 cell line was a kind gift from Dr. C. Sala (CNR Neuroscience Institute, Milan, Italy). GL261 cells were grown in complete Dulbecco’s modified Eagle’s medium (DMEM) containing 10% Newborn calf serum, 4.5 g/L glucose, 2 mM glutamine, 100 UI/mL penicillin, and 100 mg/mL streptomycin at 37 °C in 5% CO_2_ with media changes three times per week [78]. After the administration of tramadol (intraperitoneal injection, ip; 10 mg/kg), a mixture of ketamine and xylazine was used as anesthesia (ip; 100/10 mg/kg body weight). GBM-designated mice received a stereotaxically guided injection of 40,000 GL261 cells (20,000 cells/1 μL PBS solution) into the primary motor cortex (i.e., 1.75 mm lateral and 0.5 mm anterior to bregma). The GL261 cell suspension was slowly delivered at a depth of 0.8–0.9 mm from the pial surface. Body temperature was monitored with a rectal probe and maintained at 37.0 °C with a thermostat-controlled electric blanket during surgery. To facilitate breathing, an oxygen mask was placed in front of the animal’s mouth. Subcutaneous injection of saline (0.9% NaCl, 1 mL) was delivered at the end of the procedure to prevent dehydration.

#### 4.2.3. Blood Serum and CSF Sampling

For blood serum withdrawal, mice were anesthetized with isoflurane and the retro-orbital vein punctured with gentle pressure and twisting motion with a needle at the sinus level, as described by Hoggatt et al., 2018 [79]; blood was collected in 1.5 mL Eppendorf tubes and left to clot for 30 min at RT. Clotted blood was centrifuged at 2000× *g* for 20 min; serum was collected and immediately stored at -80°C. For CSF withdrawal, mice received ketamine/xylazine anesthesia (ip; 100/10 mg/kg body weight) and were placed in a stereotaxic apparatus; CSF was slowly collected from lateral ventricles (coordinates from bregma: 1 mm lateral, 0.8 mm posterior, depth of 2 mm) using a Hamilton syringe and stored at −80 °C. During CSF withdrawal, body temperature was monitored with a rectal probe and maintained at 37.0 °C with a thermostat-controlled electric blanket; an oxygen mask was placed in front of the animal to facilitate breathing and a subcutaneous injection of saline (0.9% NaCl, 1 mL) was delivered at the end of the procedure to prevent dehydration.

#### 4.2.4. Motor Tests

The grip strength test and Rotarod test were used to longitudinally evaluate motor capabilities of naïve and glioma-bearing mice, as previously described [25]. Each glioma-bearing animal performed the tests before GL261 injection (baseline measurement) and after glioma inoculation (specifically, 5, 7, 9, 12, 15, 17, 19, and 21-days post injection). Naïve mice performed the test at the same time points. All tests were performed during the same time interval each day (2:00–5:00 pm; light phase) to exclude any influence of circadian rhythms. All motor tests and data analysis were performed blind to animal treatment. Statistical analysis was performed using GraphPad Prism v5 (GraphPad Software, Inc., San Diego, CA, USA).

The grip strength test was performed by placing the animal over a base plate, in front of a grasping bar (trapezoid-shaped). The bar is linked to a force transducer connected to a Peak Amplifier (Ugo Basile S.R.L., Gemonio, Italy). When pulled by the tail, the animal instinctively grasps at the bar, until the pulling force overcomes their grip strength. The peak amplifier registers the peak pull-force achieved by the forelimbs when the animal loses its grip on the grasping bar. Three trials per day were performed for each animal and the average was calculated. All experimental values obtained were normalized to each animal’s baseline performance [25,80].

The rotarod test was performed by placing mice on a drum rotating at a baseline speed of 4 rpm (Ugo Basile S.R.L., Gemonio, Italy). The rotation speed of the drum increased linearly from 4 to 40 rpm during a 10-min observation period. An automated unit recorded the time each mouse spent on the Rotarod before falling. Each trial ended when the mouse fell from the apparatus or when 10 min had elapsed. Five consecutive trials for each mouse, with an interval of 5 min between trials, were performed. Averaged fall latency was calculated for each animal. The apparatus was cleaned with 10% ethanol to prevent the accumulation of olfactory cues. All experimental values were normalized to each animal’s baseline performance [25,81]. No difference in motor test performance was observed between male and female mice.

### 4.3. Sample Processing

#### 4.3.1. Serum

Serum samples were processed according to the protocol described in detail in Supplementary Material 1. Briefly, 15 μL of serum were immunodepleted using an Agilent MOUSE-3 spin cartridge to deplete serum albumin, serotransferrin, and IgGs. The depletion buffer was then exchanged to an SDC-based lysis buffer (0.4% SDC, 100 mM TRIS, pH 8.5, and a tablet of protease inhibitor) using a Vivaspin 500 3 kDa MWCO spin filter. Proteins were reduced and alkylated with 1 mM TCEP and 4 mM CAA. Samples were digested with 1:50 Lys-C for 4 h at 37 °C followed by 1:25 trypsin for 18 h at 37 °C. SDC was precipitated with two 12 µL aliquots of 10% FA followed by 20 min centrifugation at 20,870× *g*. The supernatant was then desalted using an AssayMAP Bravo liquid handler robot (Agilent) equipped with C18 cartridges and vacuum dried.

Peptide samples were randomized and labeled with TMT 10plex reagents using an AssayMAP Bravo system [33]. Briefly, 10 µg peptides were resuspended in a labeling buffer, loaded on RPS cartridges, and on-column labeled with a 1:15 peptide-to-TMT ratio in a two-step reaction. The labeling reaction was quenched with 2 µL of 4% hydroxylamine solution followed by 10 min of incubation at RT. Two channels of each 10-plex set were used to label a pooled sample, which served as a reference to normalize protein intensities across different LC runs [82].

The 10-plex TMT sets were mixed and then fractionated on RPS cartridges using an automated protocol on the AssayMAP BRAVO. Seven fractions (10 mM NH_4_OH in LC-MS grade water, pH 10 with 0%, 12%, 18%, 24%, 30%, 36%, and 80% of ACN, fractions 0 to 6, respectively) were collected and vacuum-dried. Dried fractions were resuspended in 10% FA. Fractions 0 and 5 and fractions 1 and 6 were pooled before injection. Approximately 1.5 μg of peptides were loaded onto an EASY-nLC 1000 (Thermo Fisher Scientific, Rockford, IL, USA) equipped with an Acclaim PepMap 100 pre-column (2 cm × 75 μm, C18, 3 μm, 100 Å; Thermo Fisher Scientific, Rockford, IL, USA). Peptides were separated on an EASY-Spray^TM^ analytical column (ES803: 50 cm × 75 µm, C18, 2 µm, 100 Å; Thermo Fisher Scientific, Rockford, IL, USA) and analyzed using an Orbitrap Fusion mass spectrometer. The MS^2^ spectra used for peptide identification were acquired in the linear ion trap (mass range 375–1500 *m*/*z*, AGC target 5.0 × 10^3^, maximum injection time 125 ms) after CID fragmentation (35% normalized collision energy). MS^3^ spectra were used for reporter-ion-quantification and were acquired in the Orbitrap (mass range 100–500 *m*/*z*, AGC target 1.0 × 10^5^, maximum injection time 150 ms) after top-eight multinotch isolation and HCD fragmentation (50% normalized collision energy).

#### 4.3.2. Serum Small Extracellular Vesicles

The serum proteome and serum sEV proteome workflows were designed so that both analyses could be performed using the 75 μL of serum available from each animal at each time point. In brief, the 50 μL serum sample was centrifuged and filtered through 0.22 μm spin filters to remove cell debris and larger vesicles. sEVs were then purified by Size Exclusion Chromatography (SEC) using qEV-70 nm columns. The elution was performed in Phosphate Saline Buffer (PBS) previously filtered with 0.2 um syringe filters and sonicated to remove air bubbles. The first three SEC-eluted fractions were pooled together and considered the sEV-pure fraction (600 μL in PBS). The sEV suspension was then loaded onto 3 kDa MWCO filters, the vesicles were rinsed with PBS, and then the buffer was exchanged to a lysis buffer (1% SDS, 5 mM EDTA, 5 mM EGTA, 10mM HEPES, pH 8.5, and protease inhibitor). The resulting sEV lysate (approximately 60 μL) was then digested using a modified SP3 protocol [33,83,84]. Specifically, the protein extract solutions were mixed with TFE in a 1:1 ratio and 2 µL of carboxylate coated paramagnetic beads (100 mg/mL suspension of 50% Speedbeads A (GE65152105050250, Sigma-Aldrich, Saint Louis, MO, USA) and 50% Speedbeads B (GE65152105050250, Sigma). Proteins were denatured at 95 °C for 5 min, then reduced with the addition of 1 μL of 200 mM DTT for each 20 μL of starting solution and alkylated with the addition of 1 μL of 400 mM IAA for each 20 μL of starting solution. Then, 50% ACN was used to promote protein adsorption to the magnetic beads, after which the bead-bound proteins were rinsed with EtOH 70% and ACN 100% and eluted in 10 μL 50mM HEPES pH 8.

The total protein concentration of each sample was determined using a modified MicroBCA assay [33]. The proteins were then digested overnight (16 h, trypsin/Lys-C digestion, 1:25 enzyme/protein) followed by a shorter supplementary step (2 h, ACN 60%—Try/Lys-C 1:75). The resulting proteolytic peptides were purified by adding 95% ACN to promote peptide binding to the beads. Peptides were rinsed and then eluted from the beads with a 2% DMSO aqueous solution. Samples were diluted 1:1 with 10% formic acid and injected into the EASY-nLC 1000 coupled to the Orbitrap Fusion mass spectrometer. Peptide ions were analyzed using the Top Speed data-dependent method, with a 3 s cycle; MS1 scans were performed in the Orbitrap (*m*/*z* 375 to 1500 at 120 K resolution with an AGC Target 5 × 10^5^ and 100 ms maximum injection time) and MS2 scans were acquired in the ion trap using a 1.6 *m*/*z* isolation window, 30% HCD Collision Energy, and an AGC target of 5 × 10^3^.

#### 4.3.3. Cerebrospinal Fluid

CSF samples were processed with the same SP3 workflow used for serum extracellular vesicles. In total, 15 µL of the SP3 lysis buffer was added to 5 µL of CSF and the resulting mixture was diluted 1:1 with TFE. Then, 2 µL of the SP3 beads suspension was added, and protein reduction, alkylation, cleanup, and quantification were carried out as described above. Proteins were digested in a single overnight step (18 h, trypsin/Lys-C digestion, 1:25 enzyme/protein). After peptide cleanup, the peptides were eluted from beads with 2% DMSO solution, diluted 1:1 with 10% FA, and injected into the nLC-MS/MS system. To increase the depth of coverage of the CSF proteome, a pooled sample was prepared by pooling five CSF peptide samples (two BL, one T1, and two T2, all of which were visibly contaminated with blood and so considered unsuitable for the longitudinal study of individual animals). The pooled sample was then subject to high pH fractionation using an AssayMAP BRAVO equipped with RPS cartridges; the fractions corresponded to isocratic elutions using 10 mM NH_4_OH in LC-MS-grade water, pH 10, with 0%, 12%, 18%, 24%, 30%, 36%, and 80% ACN. Each fraction was then analyzed using the same LC-MS conditions as the individual CSF samples. The MaxQuant match-between-runs option was used to compare individual CSF samples and to transfer identifications from the fractions to the individual CSF samples.

### 4.4. LC-MS/MS Data Analysis

Raw data of the serum dataset were analyzed using the Proteome Discoverer (v.2.1, Thermo Fisher Scientific, Rockford, IL, USA) and searched against the SwissProt *Mus Musculus* database (Uniprot, 11 June 2019, 17,021 entries). An in-house contaminant database was added to the search (250 entries). Searches were performed with a precursor mass tolerance of 10 ppm using a strict FDR of 0.01. A maximum of two missed cleavages was allowed. Methionine oxidation (+15.995 Da) and acetylation (+42.01 Da, protein *N*-terminus) were set as dynamic modifications while carbamidomethylation of cysteine (+57.021 Da) and TMT 10plex of peptide N-terminus and lysines (+229.163 Da) were set as static modifications. Protein Groups were filtered by eliminating contaminants, serum albumin, serotransferrin, and IgGs, whose intensities may depend on the depletion efficiency. Master protein intensities were exported in Excel and normalized using an in-house coded script according to Xiao et al., 2015 [82]. Specifically, the total intensity of each TMT channel was normalized to the average total intensity to correct for any variability in sample loading. TMT inter-set normalization was performed using the average of the intensities of each protein in the two normalization standard channels: The average intensities of each protein in these channels were equalized across all the TMT sets using protein-specific correction factors. PCA score plots of the serum dataset after the intra- and inter-set normalization showed that pooled samples cluster together at the center of the score plot, confirming the efficacy of the normalization method (Figure S4E,F). Protein intensities were log2 transformed prior to further analysis.

Raw data files from the CSF and sEVs experiments were processed with MaxQuant [83] (v.1.6.3.4, MaxPlanck Institute, Munich, Germany). Fully tryptic peptides were searched against the same SwissProt *Mus Musculus* protein database. The search was performed allowing a maximum of two missed cleavages, methionine oxidation (+15.995 Da) and acetylation (+42.01 Da, protein N-terminus) as dynamic modifications, and cysteine carbamidomethylation (+57.021 Da) as a static modification. For both sEV and CSF datasets, the match-between-runs method was performed (0.7 min retention time alignment) to increase the proteome coverage by using an sEV sample from three healthy mice and fractions of the pooled sample for CSF. Peptides were filtered to a minimum length of 7 amino acids and FDR was set to 0.01. Quantification was performed only on proteins identified with at least with one unique peptide. Protein intensities were used as a proxy of protein abundance. Highly variable serum proteins (serum albumin and IgGs) were filtered out from the sEV dataset, since their intensity is likely to depend on the sEV isolation efficiency. Protein intensities were log2 transformed and the median of protein intensity of each sample was subtracted from each protein intensity to correct for small differences in the number of peptides loaded on the LC-MS/MS instrument. The effect of median normalization on protein log2 intensities of the sEV and CSF datasets is shown in Figure S4A–D.

### 4.5. Longitudinal Statistical Model

The analysis of longitudinal datasets must include the time dependence of the detected protein intensities. A linear mixed effect model (LME) was fitted to the data for each protein group [44]. This model is robust to missing values [44] for some time points and is thus suitable for proteomics data, for which missing values of low-abundance proteins for some samples are common. The model includes a fixed effect for the time points and random intercepts for each individual mouse. The model for each protein is:

$$y_{k,j}{=(\beta }_{0}{+\nu }_{0,k}{)+\beta }_{1,j}\tau_{j\;}{+\varepsilon }_{k,j}$$

where the intensity y_k,j_ of each protein is predicted by the fixed effect of the time point τ_j_ and the random effect ν_0,k_ of the mouse k. The time was set as a categorical variable with three levels (baseline, T1, and T2). Both the residuals and the random effect of the model should be normally distributed [44]. The model was fitted for each protein of the dataset if the number of valid values matched the chosen conditions and if both the residuals and the random effects were normally distributed (Shapiro–Wilk test, α = 0.05). The model was tested for significant effects of the time points, and the p-values were adjusted for an FDR of 0.05 with the Benjamini–Hochberg method. Significant time point effects were plotted with their confidence interval at 95%. Regression coefficients represent the contribution of the time effect to the log2 transformed protein intensities and can be interpreted as fold changes. The same LME model was used for the serum, sEVs, and CSF datasets. Matlab (v. R2016a, MathWorks Inc., Natick, MA, USA) was used for model fitting.

Protein groups significantly upregulated or downregulated were analyzed using STRING v.11.0 [84], setting the whole *Mus Musculus* genome as the statistical background.

## 5. Conclusions

The methodologies reported here allowed the investigation of pre-symptomatic and symptomatic changes in the abundance of serum, serum sEV, and CSF proteins in a syngenic GBM mouse model, using motor tests to monitor disease progression. These longitudinal experiments revealed more than forty proteins that were dysregulated during GBM development, several of which have previously been reported as candidate diagnostic biomarkers (Vtn, Flna, C1qa, and Gsn) or prognostic biomarkers (Ahsg). Of these proteins, Gsn and Ahsg were significantly dysregulated at the pre-symptomatic stage as well as the symptomatic stage; Vtn and C1qa exhibited a dysregulation trend at the pre-symptomatic stage but were significantly different at the symptomatic stage. These findings suggest that some of the circulating proteins previously proposed as candidate GBM biomarkers could be repurposed for earlier detection. Moreover, the changes in the protein profiles of biological fluids and vesicle content suggest that GBM systemic effects can be detected before the onset of motor symptoms. The proposed sEV markers exhibited larger fold changes compared to those identified from serum, with many markers showing a dysregulation at the pre-symptomatic stage; these experiments indicate that circulating vesicles may be the most promising source of GBM biomarkers.

## Data Availability

Proteomics data have been deposited in the ProteomeXchange Consortium (http://proteomecentral.proteomexchange.org (accessed on 24 September 2021)) via the PRIDE partner repository [85] with the dataset identifier PXD020285. All the other data are available from the corresponding author.

## Supplementary Materials

The following files are available free of charge. Supplementary Material 1 (.doc). Supplementary Material 2. Quantification matrices and list of significant proteins with their regression coefficients (.xlsx). Supplementary Material 3. Enrichment analysis—Biological processes Table (.xlsx). Supplementary Material 4. Protein in common between serum and sEV datasets—Serum sEV protein groups present in the ExoCarta Top 100 database. Comparision of protein groups identified in the CSF dataset and the literature. Table (.xlsx). Figure S1. Representative images taken from a glioma-bearing mouse 23 days after tumor implantation. Scale bar = 1 mm. Figure S2. (A) Venn diagram showing the overlap between serum and serum sEV protein groups identified. (B) Venn diagram showing the comparison of the CSF proteins identified in the present study (Greco et al.) with the proteins identified in three other studies involving proteomics of CSF of murine models of brain injury and neurodegenerative diseases. Figure S3. KEGG pathways GO enrichment analysis of proteins upregulated (red) or downregulated (green) at pre-symptomatic stage. Figure S4. Box plot of log2(intensities) of proteins of sEV and CSF dataset before (A–C) and after (B–D) median normalization to correct for load effect. PCA score plot of serum samples (black: Baseline; orange: T1; red: T2; green: Pooled) before € and after (F) intra and inter set normalization. Pooled samples cluster together at the center of the score plot. Figure S5. Average of labeled, non-labeled, and overlabeled PSMs from the five TMT sets of the serum dataset. Error bars indicate standard deviation.

## Sample Availability

Please contact the authors for sample availability.

## Abbreviations

GBM, glioblastoma multiforme; CSF, cerebrospinal fluid; sEVs, small extracellular vesicles; LC, liquid chromatography; MS, mass spectrometry; Tris, tris(hydroxymethyl)aminomethane; MWCO, molecular weight cut-off; EDTA, Ethylenediaminetetraacetic acid; Lys-C, lysyl endopeptidase C; RPS, reverse phase S; TCEP, tris(2-carboxyethyl)phosphine; CAA, chloroacetamide; SDC, sodium deoxycholate; FA, formic acid; TMT, tandem mass tag; SEC, size-exclusion chromatography; PBS, phosphate-buffered saline; SDS, sodium dodecyl sulfate, EGTA, ethylene glycol-bis(β-aminoethyl ether)-N,N,N′,N′-tetraacetic acid; HEPES, 4-(2-Hydroxyethyl)piperazine-1-ethanesulfonic acid; SP3, single–pot solid-phase-enhanced sample preparation; TFE, 2,2,2-trifluoroethanol; DTT, dithiothreitol; MicroBCA, micro bicinchoninic acid assay; ACN, acetonitrile; Try, trypsin; DMSO, dimethyl sulfoxide; CID, collision-induced dissociation; HCD, higher-energy collision dissociation; NCE, normalized collision energy; PSM, peptide-spectrum match; FDR, false discovery rate; ECM, extracellular matrix.

## References

[1] Louis D.N., Perry A., Reifenberger G., Von Deimling A., Figarella D., Webster B., Hiroko K.C., Wiestler O.D., Kleihues P., Ellison D.W. (2016). The 2016 World Health Organization Classification of Tumors of the Central Nervous System: A summary. Acta Neuropathol..

[2] Parsons D.W., Jones S., Zhang X., Lin J.C., Leary R.J., Angenendt P., Mankoo P., Carter H., Siu I., Gallia G.L. (2008). An Integrated Genomic Analysis of Human Glioblastoma Multiforme. Science.

[3] Alifieris C., Trafalis D.T. (2015). Glioblastoma multiforme: Pathogenesis and treatment. Pharmacol. Ther..

[4] Chaichana K.L., Halthore A.N., Parker S.L., Olivi A., Weingart J.D., Brem H., Quinones-Hinojosa A. (2011). Factors involved in maintaining prolonged functional independence following supratentorial glioblastoma resection: Clinical article. J. Neurosurg..

[5] Gatta G., Mallone S., van der Zwan J.M., Trama A., Siesling S., Capocaccia R., Hackl M., Van Eycken E., Henau K., Hedelin G. (2014). Cancer survival in Europe 1999-2007 by country and age: Results of EUROCARE-5—A population-based study. Lancet Oncol..

[6] McPhail S., Johnson S., Greenberg D., Peake M., Rous B. (2015). Stage at diagnosis and early mortality from cancer in England. Br. J. Cancer.

[7] Sant M., Allemani C., Capocaccia R., Hakulinen T., Aareleid T., Coebergh J.W., Coleman M.P., Grosclaude P., Martinez C., Bell J. (2003). Stage at diagnosis is a key explanation of differences in breast cancer survival across Europe. Int. J. Cancer.

[8] Schröder F.H., Hugosson J., Roobol M.J., Tammela T.L.J., Zappa M., Nelen V., Kwiatkowski M., Lujan M., Määttänen L., Lilja H. (2014). Screening and prostate cancer mortality: Results of the European Randomised Study of Screening for Prostate Cancer (ERSPC) at 13 years of follow-up. Lancet.

[9] Cuzick J., Thorat M.A., Andriole G., Brawley O.W., Brown P.H., Culig Z., Eeles R.A., Ford L.G., Hamdy F.C., Holmberg L. (2014). Prevention and early detection of prostate cancer. Lancet Oncol..

[10] Sauzay C., Petit A., Bourgeois A.M., Barbare J.C., Chauffert B., Galmiche A., Houessinon A. (2016). Alpha-foetoprotein (AFP): A multi-purpose marker in hepatocellular carcinoma. Clin. Chim. Acta.

[11] Zhang J., Chen G., Zhang P., Zhang J., Li X., Gan D., Cao X., Han M., Du H., Ye Y. (2020). The threshold of alpha-fetoprotein (AFP) for the diagnosis of hepatocellular carcinoma: A systematic review and meta-analysis. PLoS ONE.

[12] Wu G., Wu J., Wang B., Zhu X., Shi X., Ding Y. (2018). Importance of tumor size at diagnosis as a prognostic factor for hepatocellular carcinoma survival: A population-based study. Cancer Manag. Res..

[13] Marrero J.A., Feng Z., Wang Y., Nguyen M.H., Befeler A.S., Roberts L.R., Reddy K.R., Harnois D., Llovet J.M., Normolle D. (2009). α-Fetoprotein, Des-γ Carboxyprothrombin, and Lectin-Bound α-Fetoprotein in Early Hepatocellular Carcinoma. Gastroenterology.

[14] Sole C., Arnaiz E., Manterola L., Otaegui D., Lawrie C.H. (2019). The circulating transcriptome as a source of cancer liquid biopsy biomarkers. Semin. Cancer Biol..

[15] Han X., Wang J., Sun Y. (2017). Circulating Tumor DNA as Biomarkers for Cancer Detection. Genom. Proteom. Bioinforma.

[16] Stromberg L.R., Lilley L.M., Mukundan H. (2019). Advances in lipidomics for cancer biomarker discovery. Proteomic and Metabolomic Approaches to Biomarker Discovery.

[17] Belczacka I., Latosinska A., Metzger J., Marx D., Vlahou A., Mischak H., Frantzi M. (2019). Proteomics biomarkers for solid tumors: Current status and future prospects. Mass Spectrom. Rev..

[18] Armitage E.G., Southam A.D. (2016). Monitoring cancer prognosis, diagnosis and treatment efficacy using metabolomics and lipidomics. Metabolomics.

[19] Touat M., Duran-Peña A., Alentorn A., Lacroix L., Massard C., Idbaih A. (2015). Emerging circulating biomarkers in glioblastoma: Promises and challenges. Expert Rev. Mol. Diagn..

[20] Stylli S.S., Luwor R.B., Ware T.M.B., Tan F., Kaye A.H. (2015). Mouse models of glioma. J. Clin. Neurosci..

[21] Newcomb E.W., Zagzag D., Van Meir E.G. (2009). The Murine GL261 Glioma Experimental Model to Assess Novel Brain Tumor Treatments. Cancer Drug Discovery and Development.

[22] Diehl K.H., Hull R., Morton D., Pfister R., Rabemampianina Y., Smith D., Vidal J.M., Van De Vorstenbosch C. (2001). A good practice guide to the administration of substances and removal of blood, including routes and volumes. J. Appl. Toxicol..

[23] (2019). Animal Research Advisory Committee—Office of Animal Care Guidelines for Blood Collection in Mice and Rats.

[24] Larjavaara S., Mäntylä R., Salminen T., Haapasalo H., Raitanen J. (2007). Incidence of gliomas by anatomic location. Neurooncology.

[25] Vannini E., Maltese F., Olimpico F., Fabbri A., Costa M., Caleo M., Baroncelli L. (2017). Progression of motor deficits in glioma-bearing mice: Impact of CNF1 therapy at symptomatic stages. Oncotarget.

[26] Echan L.A., Tang H.Y., Ali-Khan N., Lee K.B., Speicher D.W. (2005). Depletion of multiple high-abundance proteins improves protein profiling capacities of human serum and plasma. Proteomics.

[27] Fountoulakis M., Juranville J.F., Jiang L., Avila D., Röder D., Jakob P., Berndt P., Evers S., Langen H. (2004). Depletion of the high-abundance plasma proteins. Amino Acids.

[28] Gardiner C., Di Vizio D., Sahoo S., Théry C., Witwer K.W., Wauben M., Hill A.F. (2016). Techniques used for the isolation and characterization of extracellular vesicles: Results of a worldwide survey. J. Extracell. Vesicles.

[29] Ramirez M.I., Amorim M.G., Gadelha C., Milic I., Welsh J.A., Freitas V.M., Nawaz M., Akbar N., Couch Y., Makin L. (2018). Technical challenges of working with extracellular vesicles. Nanoscale.

[30] Kim J., Tan Z., Lubman D.M. (2015). Exosome enrichment of human serum using multiple cycles of centrifugation. Electrophoresis.

[31] Doyle L.M., Wang M.Z. (2019). Overview of Extracellular Vesicles, Their Origin, Composition, Purpose, and Methods for Exosome Isolation and Analysis. Cells.

[32] Anastasi F., Greco F., Dilillo M., Vannini E., Cappello V., Baroncelli L., Costa M., Gemmi M., Caleo M., McDonnell L.A. (2020). Proteomics analysis of serum small extracellular vesicles for the longitudinal study of a glioblastoma multiforme mouse model. Sci. Rep..

[33] De Graaf E.L., Pellegrini D., McDonnell L.A. (2016). Set of Novel Automated Quantitative Microproteomics Protocols for Small Sample Amounts and Its Application to Kidney Tissue Substructures. J. Proteome Res..

[34] Sleat D.E., Wiseman J.A., El-Banna M., Zheng H., Zhao C., Soherwardy A., Moore D.F., Lobel P. (2019). Analysis of brain and cerebrospinal fluid from mouse models of the three major forms of neuronal ceroid lipofuscinosis reveals changes in the lysosomal proteome. Mol. Cell. Proteom..

[35] Hsu W.H., Shen Y.C., Shiao Y.J., Kuo C.H., Lu C.K., Lin T.Y., Ku W.C., Lin Y.L. (2019). Combined proteomic and metabolomic analyses of cerebrospinal fluid from mice with ischemic stroke reveals the effects of a Buyang Huanwu decoction in neurodegenerative disease. PLoS ONE.

[36] Dislich B., Wohlrab F., Bachhuber T., Müller S.A., Kuhn P.H., Hogl S., Meyer-Luehmann M., Lichtenthaler S.F. (2015). Label-free quantitative proteomics of mouse cerebrospinal fluid detects β-site APP cleaving enzyme (BACE1) protease substrates in vivo. Mol. Cell. Proteom..

[37] Liu S., Ji W., Lu J., Tang X., Guo Y., Ji M., Xu T., Gu W., Kong D., Shen Q. (2020). Discovery of Potential Serum Protein Biomarkers in Ankylosing Spondylitis Using Tandem Mass Tag-Based Quantitative Proteomics. J. Proteome Res..

[38] Liu H., Chen H., Wu X., Sun Y., Wang Y., Zeng Y., Chen G., Liu X., Xing X., Zhao B. (2019). The serum proteomics tracking of hepatocellular carcinoma early recurrence following radical resection. Cancer Manag. Res..

[39] Zhang Y., Guo Z., Zou L., Yang Y., Zhang L., Ji N., Shao C., Sun W., Wang Y. (2015). A comprehensive map and functional annotation of the normal human cerebrospinal fluid proteome. J. Proteom..

[40] Guldbrandsen A., Vethe H., Farag Y., Oveland E., Garberg H., Berle M., Myhr K.M., Opsahl J.A., Barsnes H., Berven F.S. (2014). In-depth characterization of the cerebrospinal fluid (CSF) proteome displayed through the CSF proteome resource (CSF-PR). Mol. Cell. Proteomics.

[41] Schutzer S.E., Liu T., Natelson B.H., Angel T.E., Schepmoes A.A., Purvine S.O., Hixson K.K., Lipton M.S., Camp D.G., Coyle P.K. (2010). Establishing the proteome of normal human cerebrospinal fluid. PLoS ONE.

[42] Barkovits K., Linden A., Galozzi S., Schilde L., Pacharra S., Mollenhauer B., Stoepel N., Steinbach S., May C., Uszkoreit J. (2018). Characterization of Cerebrospinal Fluid via Data-Independent Acquisition Mass Spectrometry. J. Proteome Res..

[43] Lin L., Zheng J., Yu Q., Chen W., Xing J., Chen C., Tian R. (2018). High throughput and accurate serum proteome profiling by integrated sample preparation technology and single-run data independent mass spectrometry analysis. J. Proteom..

[44] Hedeker D., Gibbons R.D., Balding D.J., Cressie N.A.C., Fisher N.I., Johnstone I.M., Kadane J.B., Geert M., Ryan L.M., Scott D.W., Smith A.F.M., Teugels J.L. (2006). Mixed-Effects regression models for continuous outcomes. Longitudinal Data Analysis.

[45] Carlsson A.C., Ingelsson E., Sundström J., Carrero J.J., Gustafsson S., Feldreich T., Stenemo M., Larsson A., Lind L., Ärnlöv J. (2017). Use of proteomics to investigate kidney function decline over 5 years. Clin. J. Am. Soc. Nephrol..

[46] Liu C.W., Bramer L., Webb-Robertson B.J., Waugh K., Rewers M.J., Zhang Q. (2017). Temporal profiles of plasma proteome during childhood development. J. Proteom..

[47] Mallawaaratchy D.M., Hallal S., Russell B., Ly L., Ebrahimkhani S., Wei H., Christopherson R.I., Buckland M.E., Kaufman K.L. (2017). Comprehensive proteome profiling of glioblastoma-derived extracellular vesicles identifies markers for more aggressive disease. J. Neurooncol..

[48] Naryzhny S., Volnitskiy A., Kopylov A., Zorina E., Kamyshinsky R., Bairamukov V., Garaeva L., Shlikht A., Shtam T. (2020). Proteome of Glioblastoma-Derived Exosomes as a Source of Biomarkers. Biomedicines.

[49] García-Romero N., Carrión-Navarro J., Esteban-Rubio S., Lázaro-Ibáñez E., Peris-Celda M., Alonso M.M., Guzmán-De-Villoria J., Fernández-Carballal C., de Mendivil A.O., García-Duque S. (2017). DNA sequences within glioma-derived extracellular vesicles can cross the intact blood-brain barrier and be detected in peripheral blood of patients. Oncotarget.

[50] Jung C.S., Unterberg A.W., Hartmann C. (2011). Diagnostic markers for glioblastoma. Histol. Histopathol..

[51] Schvartz I., Seger D., Shaltiel S. (1999). Molecules in focus: Vitronectin. Int. J. Biochem. Cell Biol..

[52] Uhm J.H., Dooley N.P., Kyritsis A.P., Rao J.S., Gladson C.L. (1999). Vitronectin, a glioma-derived extracellular matrix protein, protects tumor cells from apoptotic death. Clin. Cancer Res..

[53] Fukushima Y., Tamura M., Nakagawa H., Itoh K. (2008). Induction of glioma cell migration by vitronectin in human serum and cerebrospinal fluid. J. Neurosurg..

[54] Liu Z., Han L., Dong Y., Tan Y., Li Y., Zhao M. (2015). EGFRvIII/integrin β3 interaction in hypoxic and vitronectin- enriching microenvironment promote GBM progression and metastasis. Oncotarget.

[55] Chen M.H., Lu C., Sun J., Chen X.D., Dai J.X., Cai J.Y., Chen X.L. (2016). Diagnostic and prognostic value of serum vitronectin levels in human glioma. J. Neurol. Sci..

[56] Alper Ö., Stetler-Stevenson W.G., Harris L.N., Leitner W.W., Özdemirli M., Hartmann D., Raffeld M., Abu-Asab M., Byers S., Zhuang H. (2009). Novel anti-filamin-A antibody detects a secreted variant of filamin-A in plasma from patients with breast carcinoma and high-grade astrocytoma. Cancer Sci..

[57] Chantaravisoot N., Wongkongkathep P., Loo J.A., Mischel P.S., Tamanoi F. (2015). Significance of filamin A in mTORC2 function in glioblastoma. Mol. Cancer.

[58] Reiss Y., Scholz A., Plate K.H., Schmidt M.H., Liebner S. (2015). The Angiopoietin—Tie System: Common Signaling Pathways for Angiogenesis, Cancer, and Inflammation. Endothelial Signaling in Development and Disease.

[59] de Vega S., Kondo A., Suzuki M., Arai H., Jiapaer S., Sabit H., Nakada M., Ikeuchi T., Ishijima M., Arikawa-Hirasawa E. (2019). Fibulin-7 is overexpressed in glioblastomas and modulates glioblastoma neovascularization through interaction with angiopoietin-1. Int. J. Cancer.

[60] Armstrong L.C., Bornstein P. (2003). Thrombospondins 1 and 2 function as inhibitors of angiogenesis. Matrix Biol..

[61] Daubon T., Léon C., Clarke K., Andrique L., Salabert L., Darbo E., Pineau R., Guérit S., Maitre M., Dedieu S. (2019). Deciphering the complex role of thrombospondin-1 in glioblastoma development. Nat. Commun..

[62] Dickinson R.J., Keyse S.M. (2006). Diverse physiological functions for dual-specificity MAP kinase phosphatases. J. Cell Sci..

[63] Zhang Y.L., Wang R.C., Cheng K., Ring B.Z., Su L. (2017). Roles of Rap1 signaling in tumor cell migration and invasion. Cancer Biol. Med..

[64] Deininger M.H., Meyermann R., Schluesener H.J. (2003). Expression and release of CD14 in astrocytic brain tumors. Acta Neuropathol..

[65] Sarma J.V., Ward P.A. (2011). The complement system. Cell Tissue Res..

[66] Afshar-Kharghan V. (2017). The role of the complement system in cancer. J. Clin. Investig..

[67] Reis E.S., Mastellos D.C., Ricklin D., Mantovani A., Lambris J.D. (2018). Complement in cancer: Untangling an intricate relationship. Nat. Rev. Immunol..

[68] Bouwens T.A.M., Trouw L.A., Veerhuis R., Dirven C.M.F., Lamfers M.L.M., Al-Khawaja H. (2015). Complement activation in Glioblastoma Multiforme pathophysiology: Evidence from serum levels and presence of complement activation products in tumor tissue. J. Neuroimmunol..

[69] Petrik V., Saadoun S., Loosemore A., Hobbs J., Opstad K.S., Sheldon J., Tarelli E., Howe F.A., Bell B.A., Papadopoulos M.C. (2008). Serum alpha 2 -HS Glycoprotein Predicts Survival in Patients with Glioblastoma. Clin. Chem..

[70] Miyauchi E., Furuta T., Ohtsuki S., Tachikawa M., Uchida Y., Sabit H., Obuchi W., Baba T., Watanabe M., Terasaki T. (2018). Identification of blood biomarkers in glioblastoma by SWATH mass spectrometry and quantitative targeted absolute proteomics. PLoS ONE.

[71] Ohnishi M., Matsumoto T., Nagashio R., Kageyama T., Utsuki S., Oka H., Okayasu I., Sato Y. (2009). Proteomics of tumor-specific proteins in cerebrospinal fluid of patients with astrocytoma : Usefulness of gelsolin protein. Pathol. Int..

[72] Hamson E.J., Keane F.M., Tholen S., Schilling O., Gorrell M.D. (2014). Understanding fibroblast activation protein (FAP): Substrates, activities, expression and targeting for cancer therapy. Proteom. Clin. Appl..

[73] Inda M. (2014). del M.; Bonavia, R.; Seoane, J. Glioblastoma multiforme:A look inside its heterogeneous nature. Cancers.

[74] Friedmann-Morvinski D. (2014). Glioblastoma heterogeneity and cancer cell plasticity. Crit. Rev. Oncog..

[75] Becker A., Thakur B.K., Weiss J.M., Kim H.S., Peinado H., Lyden D. (2016). Extracellular Vesicles in Cancer: Cell-to-Cell Mediators of Metastasis. Cancer Cell.

[76] Jabalee J., Towle R., Garnis C. (2018). The Role of Extracellular Vesicles in Cancer: Cargo, Function, and Therapeutic Implications. Cells.

[77] Lane R.E., Korbie D., Hill M.M., Trau M. (2018). Extracellular vesicles as circulating cancer biomarkers: Opportunities and challenges. Clin. Transl. Med..

[78] Vannini E., Panighini A., Cerri C., Fabbri A., Lisi S., Pracucci E., Benedetto N., Vannozzi R., Fiorentini C., Caleo M. (2014). The bacterial protein toxin, cytotoxic necrotizing factor 1 (CNF1) provides long-term survival in a murine glioma model. BMC Cancer.

[79] Hoggatt J., Singh P., Tate T.A., Chou B.K., Datari S.R., Fukuda S., Liu L., Kharchenko P.V., Schajnovitz A., Baryawno N. (2018). Rapid Mobilization Reveals a Highly Engraftable Hematopoietic Stem Cell. Cell.

[80] Flis D.J., Dzik K., Kaczor J.J., Cieminski K., Halon-Golabek M., Antosiewicz J., Wieckowski M.R., Ziolkowski W. (2019). Swim training modulates mouse skeletal muscle energy metabolism and ameliorates reduction in grip strength in a mouse model of amyotrophic lateral sclerosis. Int. J. Mol. Sci..

[81] Brooks S.P., Dunnett S.B. (2009). Tests to assess motor phenotype in mice: A user’s guide. Nat. Rev. Neurosci..

[82] Xiao Z., Chang J., Hendriks I.a., Sigursson J., Olsen J.V., Vertegaal A.C.O. (2017). Extended multiplexing of TMT labeling reveals age and high fat diet specific proteome changes in mouse epididymal adipose tissue. Mol. Cell. Proteom..

[83] Hughes C.S., Foehr S., Garfield D.A., Furlong E.E., Steinmetz L.M., Krijgsveld J. (2014). Ultrasensitive proteome analysis using paramagnetic bead technology. Mol. Syst. Biol..

[84] Pellegrini D., del Grosso A., Angella L., Giordano N., Dilillo M., Tonazzini I., Caleo M., Cecchini M., McDonnell L.A. (2019). Quantitative Microproteomics Based Characterization of the Central and Peripheral Nervous System of a Mouse Model of Krabbe Disease. Mol. Cell. Proteom..

[85] Cox J., Mann M. (2008). MaxQuant enables high peptide identification rates, individualized p.p.b.-range mass accuracies and proteome-wide protein quantification. Nat. Biotechnol..

