# Supplementary material for: Longitudinal Bottom-Up Proteomics of Serum, Serum Extracellular Vesicles, and Cerebrospinal Fluid Reveals Candidate Biomarkers for Early Detection of Glioblastoma in a Murine Model
Source: Molecules. 2021 Oct 2;26(19):5992. doi: 10.3390/molecules26195992 (PMC8512455; doi:10.3390/molecules26195992)
Supplement: Supplementary file 1 [file molecules-26-05992-s001.zip › SuppMat_Greco2021.pdf]

Longitudinal bottom-up proteomics of serum, serum exosomes, and cerebrospinal fluid reveals candidate biomarkers for early detection of glioblastoma in a murine model

Francesco Greco[1,2], Federica Anastasi[3,2], Luca Fidia Pardini[4,2], Marialaura Dilillo[2], Eleonora Vannini[5,6], Laura Baroncelli[5,7], Matteo Caleo[5,8] and Liam A. McDonnell[2,*]

[1] Institute of Life Sciences, Sant'Anna School of Advanced Studies, 56127 Pisa, Italy;
[2] Fondazione Pisana per la Scienza ONLUS, 56017 San Giuliano Terme (PI), Italy;
[3] NEST laboratories, Scuola Normale Superiore, 56127 Pisa, Italy;
[4] Department of Chemistry and Industrial Chemistry, University of Pisa, 56124 Pisa, Italy;
[5] CNR, Neuroscience Institute, 56124 Pisa, Italy;
[6] Fondazione Umberto Veronesi, 20122 Milano, Italy;
[7] IRCCS Fondazione Stella Maris, 56018 Calambrone (PI), Italy;
[8] Dipartimento di Scienze Biomediche, Università di Padova, 35131 Padova, Italy;
* Correspondence: liam@fpscience.it;

Supplementary Material

Serum processing

Depletion of high-abundance proteins

Depletion of high-abundance proteins was performed using the Agilent MOUSE-3 spin cartridge and vendor depletion buffers A and B. The cartridge depletes serum albumin, serotransferrin and IgGs. Serum samples were thawed on ice and 15 µL of serum diluted to 200 µL with depletion buffer A, loaded onto 0.22 µm spin filters and centrifuged at 16 000xg for 1 minute to remove cell debris. Samples were depleted according to the manufacturer's instruction. Briefly, the cartridge was equilibrated with 4 mL of depletion buffer A then mounted onto a screw-top vial and loaded with a filtered serum sample. The cartridge was then centrifuged for 2 minutes at 100xg. 400 µL of depletion buffer A were loaded onto the bed and the cartridge was further centrifuged at 100xg for 2 minutes. The flow through was collected. The cartridge was transferred to a second screw top vial, 400 µL of depletion buffer A added and the cartridge centrifuged again at 100xg for 2 minutes to collect a second flow through. All the centrifugation steps were performed at 4°C. The cartridge was regenerated with 2 mL of depletion buffer B between sample processing.

Buffer exchange

Buffer was exchanged to a 0.4% SDC solution in 100mM TRIS, pH 8.5 using 3kDa MWCO filters. A tablet of protease inhibitor was added each 10 mL of lysis buffer. All the centrifugation steps were performed at 4°C and 15 000xg. The first depletion flow through was loaded onto the filter and centrifuged for 1 hour; then the second flow through was loaded onto the filter and centrifuged for 2 hours. SDC lysis buffer was loaded in three aliquots of 200 µL, 100 µL and 100 µL, followed by 1 hour of centrifugation each. Filters were sonicated using a BioRuptor for 5 minutes (30 seconds ON, 30 seconds OFF cycles, 4°C). The solution retained on the top of the filter was transferred on a LoBind 0.5 mL Eppendorf tube and stored at -20°C overnight.

Reduction, alkylation, digestion and desalting

Serum samples were thawed on ice the following day and quantified with a MicroBCA assay. Reduction and alkylation were performed in the same step by adding a TCEP solution to a final concentration of 1 mM and a CAA to a final concentration of 4 mM and incubating at 95°C for 5 minutes. Proteins were digested with 1:50 Lys-C at 37°C for 4 hours followed by 1:30 trypsin at 37°C for 18 hours. SDC was precipitated by addition of 12 µL of a 10% FA solution followed by 20 minutes of centrifugation at 20870xg at 4°C and the supernatant collected. SDC precipitation was performed twice. Samples were then desalted using an AssayMAP BRAVO liquid handler robot. Briefly, samples were diluted with HPLC water to 110 µL, loaded onto 5 µL C18 cartridges (previously primed with ACN 0.1% FA) and washed with LC-MS water 0.1% FA. After washing with water 0.1% FA, samples were eluted with 25 µL of 80:20 ACN:LC-MS water 0.1% FA and vacuum dried.

On-column TMT labeling, fractionation and LC-MS/MS method

Serum peptides were labelled using 10plex TMT labels. Serum samples were randomly assigned to five 10plex-TMT sets. A pool of digested peptides from all samples was labelled with channels 130C and 131 and used for inter-set normalization. A protocol for automated on-column TMT labeling using the AssayMAP BRAVO was adapted for serum samples. Serum peptides were resuspended in a labeling buffer (50 mM NaH_2PO_4 in HPLC water, pH 4.5) with 5% ACN and quantified with a MicroBCA assay. Peptide quantification was used to equalize peptide loading on RPS cartridges (10 µg peptides per cartridge). Peptides were labelled with 150 µg TMT reagent delivered in two reaction steps of 30 minutes (20 µL of TMT in labeling buffer, 1 µL/min followed by 10 minutes of RT incubation for each step). Labelled peptides were eluted with 25 µL of 80:20 ACN:LC-MS water 0.1% FA into an eppendorf plate containing 2 µL of a 4% hydroxylamine solution, and incubated for 10 minutes at RT to quench the labeling reaction. 1 µg of labelled peptides was pooled for each TMT set and analyzed with an MS2 method; average reporter ion intensities of each channel were used to equalize mixing ratios of channels for each set. Labelled peptides of each set were mixed and vacuum

dried. TMT pools were high-pH fractionated on RPS cartridges using the AssayMAP BRAVO robot. Seven fractions (F0-F6, 10 mM NH_4OH in LC-MS water with 0%, 12%, 18%, 24%, 30%, 36% and 80% ACN, pH 10) were produced for each set; fractions 0 - 5, and 1 - 6 were pooled and analyzed together. Fractions were resuspended in 10% FA and 1.5 µg were injected into an EASY-nLC 1000 equipped with an Acclaim PepMap 100 pre-column (75 µm x 2 cm) packed with 3 µm C18 particles (pore size 100 Å). Peptides were separated on an Easy-Spray column (ES803, 75 µm x 50 cm, 3 µm particle size, 100 Å pore size), using LC-MS water 0.1% FA as buffer A and ACN 0.1% as buffer B. The LC gradient for serum analysis is reported in **Table S1**. The nLC was interfaced with an Orbitrap Fusion Tribrid mass spectrometer though a nESI EASY-Spray source. Full MS scans were acquired using the Orbitrap analyzer (positive polarity, resolution 120 000, scan range 375-1500 m/z, AGC target 2.00E+05 and maximum injection time 50 ms). MS2 spectra were acquired in the linear ion trap (Rapid mode, mass range 375-1500 m/z, AGC target 5.0x103, maximum injection time 125 ms) after CID fragmentation (35% NCE). MS3 spectra were acquired in the Orbitrap (top-8 multinotch isolation, HCD fragmentation, 50% NCE, mass range 100-500 m/z, AGC target 1.0x105, maximum injection time 150 ms).

TMT labeling efficiency

To evaluate TMT labeling efficiency 1 ug of labelled peptides from each TMT channel was pooled and injected. TMT labeling efficiency was performed separately for each set. The database search was performed with TMT labeling at the N-terminus and lysines set as dynamic modifications; PSMs were filtered by the modifications and the proportion of N-terminus-labeled PSMs calculated. The percentage of TMT-labeled lysines was calculated using only PSMs containing a lysine residue. The percentage of non-labelled PSMs was evaluated as the ratio between the number of PSMs not having an N-terminus modification nor a lysine modification to the total number of PSMs.

TMT overlabeling of histidine, serine, threonine and tyrosine was evaluated by setting the N-terminus and lysine labeling as static modifications and the other amino acid modifications as dynamic. The

percentage of overlabeled amino acids was evaluated by calculating the ratio between the filtered PSMs carrying an overlabeling TMT modification and the total number of PSMs. **Figure S1** shows a summary of the percentage of labeled, non labeled and overlabeled PSMs.

nLC-MS/MS method for serum sEVs

1 ug of peptides from serum sEVs were separated on an EASY-SprayTM analytical column (ES803: 50 cm x 75 µm, C18, 2 µm, 100 Å; Thermo Scientific) mounted on an EASY-nLC 1000 using the gradient specified in **Table S2**. The nLC was coupled to an Orbitrap Fusion Tribrid mass spectrometer though a nESI EASY-Spray source. Peptides were analyzed using a Top Speed method. Full MS spectra were acquired in the Orbitrap analyzer (positive polarity, resolution 120 000, scan range 375-1500 m/z, AGC target 4.0x105 and maximum injection time 100 ms). Precursor ions selected for fragmentation were isolated using the quadrupole (1.6 m/z isolation window) and fragmented using HCD (NCE 30%); the resulting fragment ions were then analyzed in the linear ion trap (Rapid mode, AGC target 1.0x104, maximum injection time 35 ms).

nLC-MS/MS method for CSF

The whole digests resulting from each CSF sample was separated on the EASY-nLC 1000 with the gradient specified in **Table S3**. The nLC system was coupled to the Orbitrap Fusion Tribrid through a nESI EASY-Spray source. Peptides were analyzed using a Top Speed method. Full MS spectra were acquired on positive mode in the Orbitrap (resolution 120 000, scan range 375-1500 m/z, AGC target 4.0x105, maximum injection time 100 ms). Ions were selected and fragmented (quadrupole selection, 1.6 m/z isolation window, HCD fragmentation, NCE 27%) and analyzed in the ion trap (Rapid mode, AGC target 2.0x10^{3}, maximum injection time 300 ms).

Table S1. nLC gradient for serum analysis.

Time (min)	Flow (nL/min)	% B
**0.00**	300	8
**01.00**	300	8
**105.00**	300	25
**120.00**	300	35
**130.00**	300	90
**140.00**	300	90

Table S2. nLC gradient for serum sEV analysis.

Time (min)	Flow (nL/min)	% B
**0.00**	300	6
**01.00**	300	6
**53.00**	300	23
**60.00**	300	33
**66.00**	300	90
**75.00**	300	90

Table S3. nLC gradient for CSF analysis.

Time (min)	Flow (nL/min)	% B
**0.00**	300	5
**01.00**	300	5
**105.00**	300	22
**120.00**	300	32
**130.00**	300	95
**145.00**	300	95

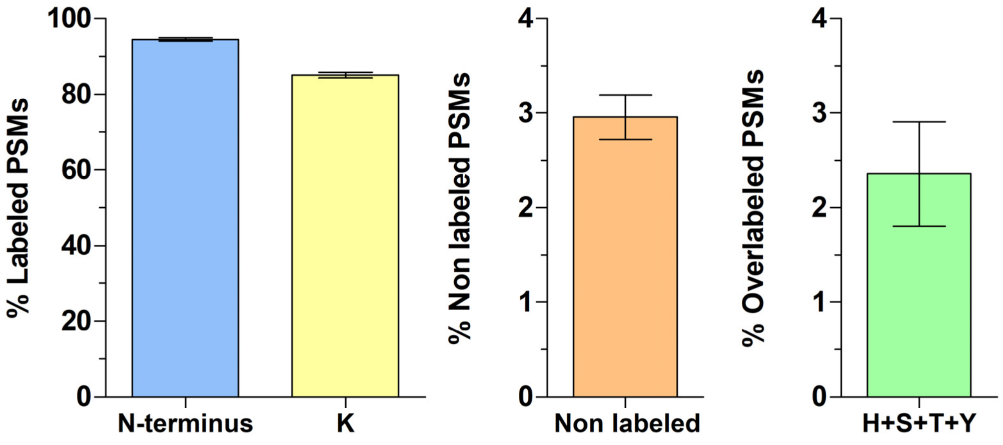

Figure S1. Average of labeled, non labeled and overlabeled PSMs from the five TMT sets of the serum dataset. Error bars indicate standard deviation.

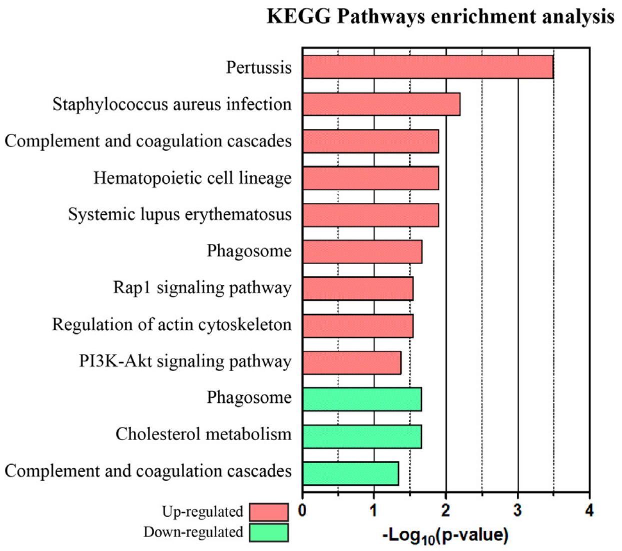

Figure S2. KEGG pathways GO enrichment analysis of proteins upregulated (red) or downregulated (green) at presymptomatic stage.

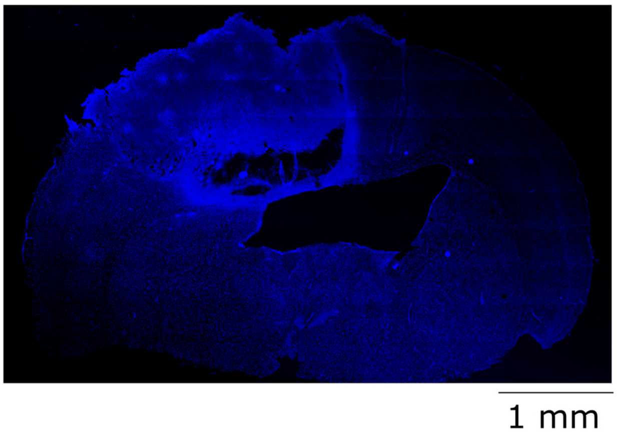

Figure S3. Representative images taken from a glioma-bearing mouse 23 days after tumor implantation. Scale bar = 1 mm.

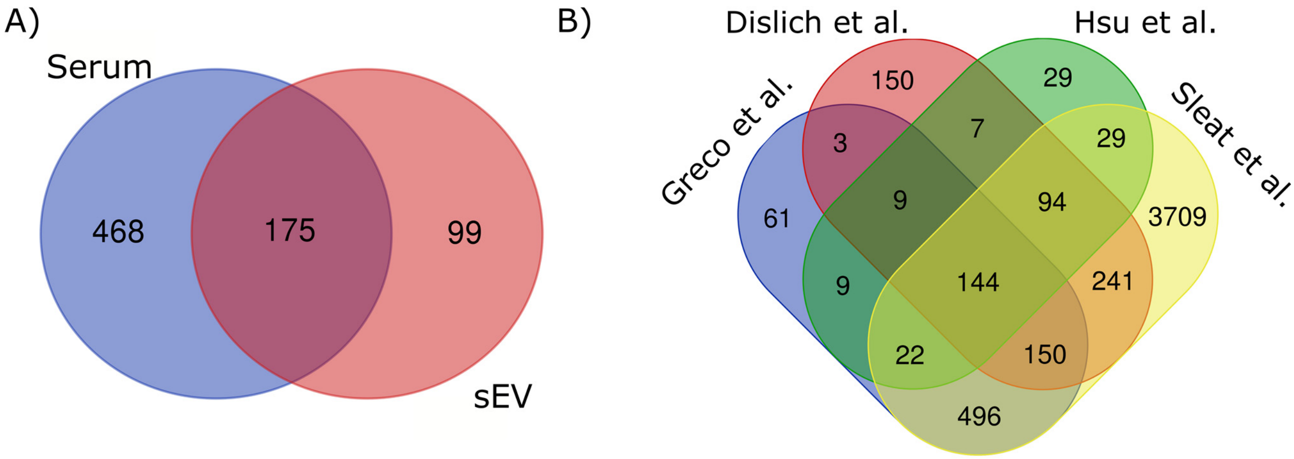

Figure S4. A) Venn diagram showing the overlap between serum and serum sEV protein groups identified. B) Venn diagram showing the comparison of the CSF proteins identified in the present study (Greco et al.) with the proteins identified in three other studies involving proteomics of CSF of murine models of brain injury and neurodegenerative diseases.

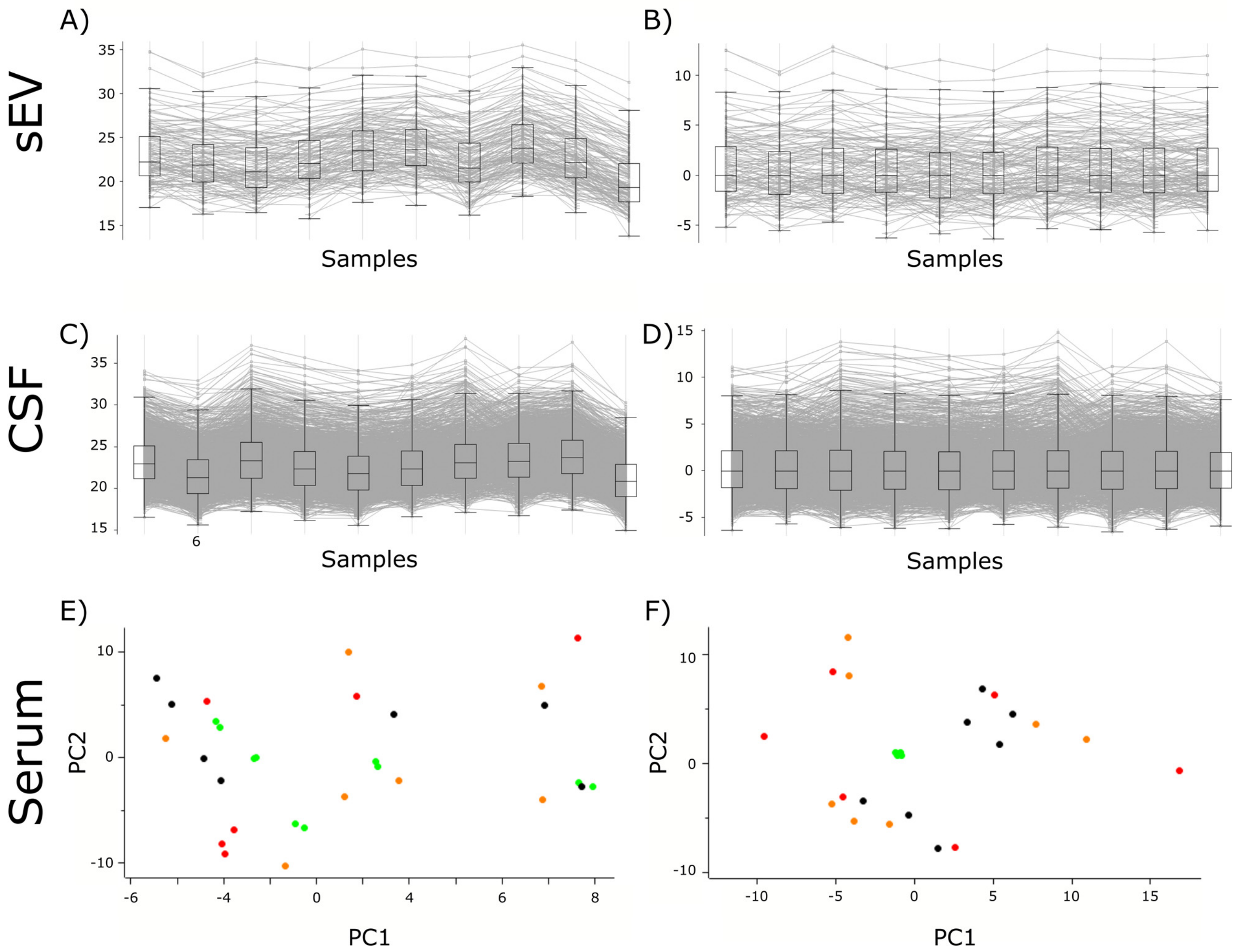

Figure S5. Box plot of log2(intensities) of proteins of sEV and CSF dataset before (A-C) and after (B-D) median normalization to correct for load effect. PCA score plot of serum samples (black: baseline; orange: T1; red: T2; green: pooled) before (E) and after (F) intra and inter set normalization. Pooled samples cluster together at the center of the score plot.
