# Supplementary figures and images for: Longitudinal Bottom-Up Proteomics of Serum, Serum Extracellular Vesicles, and Cerebrospinal Fluid Reveals Candidate Biomarkers for Early Detection of Glioblastoma in a Murine Model
Source: Molecules. 2021 Oct 2;26(19):5992. doi: 10.3390/molecules26195992 (PMC8512455; doi:10.3390/molecules26195992)

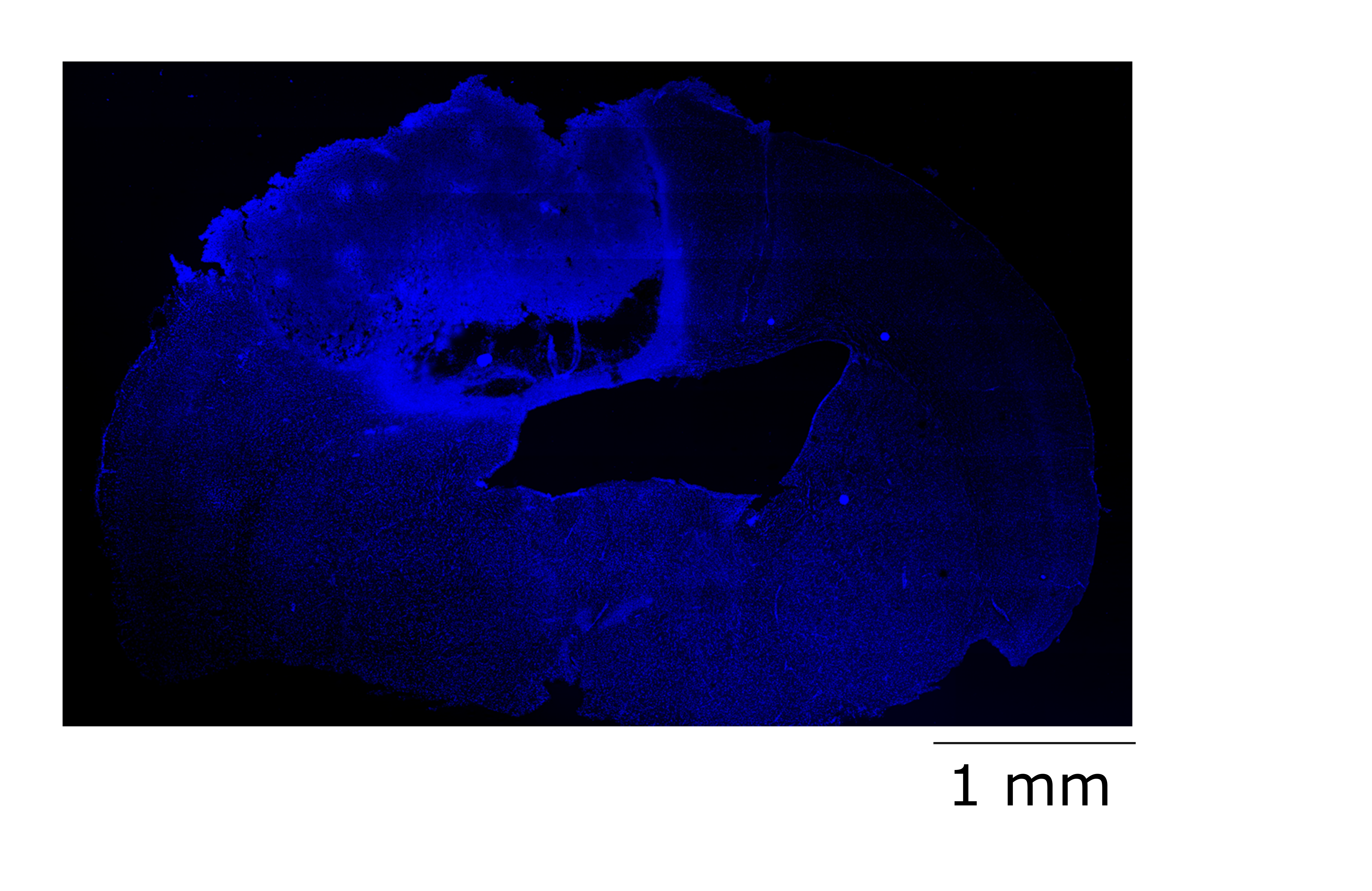

Supplement: Supplementary file 1 [file molecules-26-05992-s001.zip › Figure S1_Greco_2021.png]

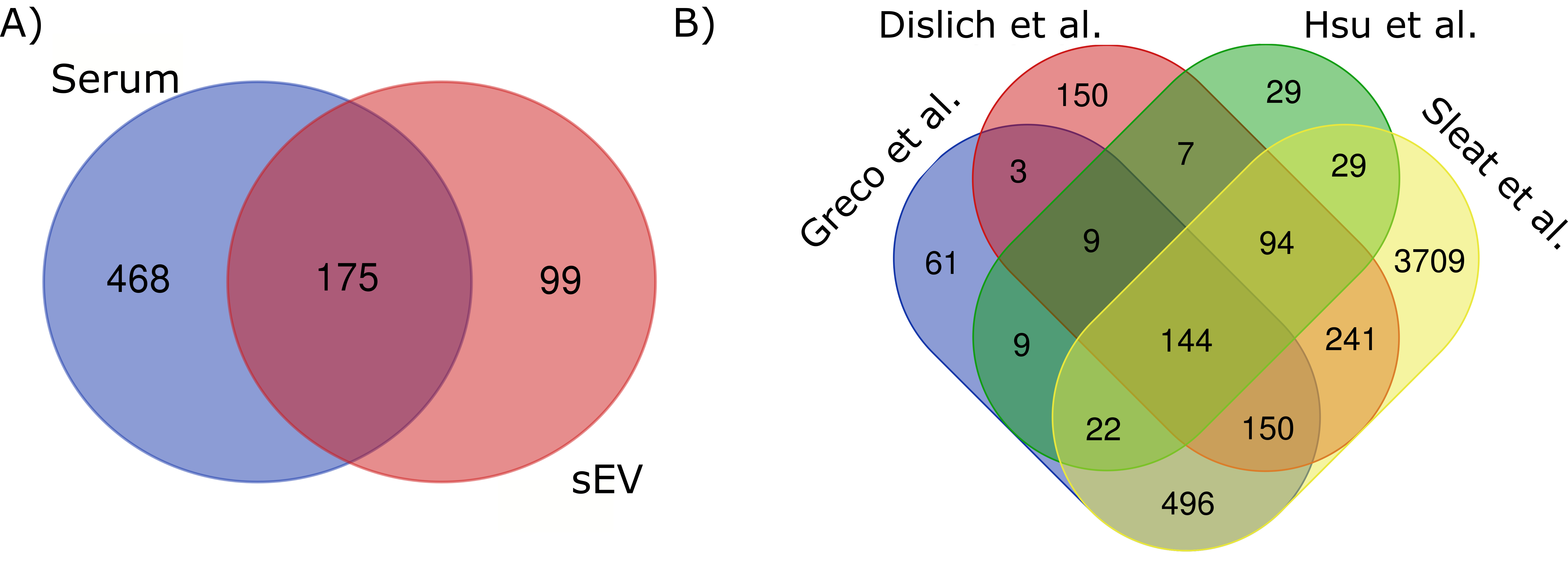

Supplement: Supplementary file 1 [file molecules-26-05992-s001.zip › Figure S2_Greco_2021.png]

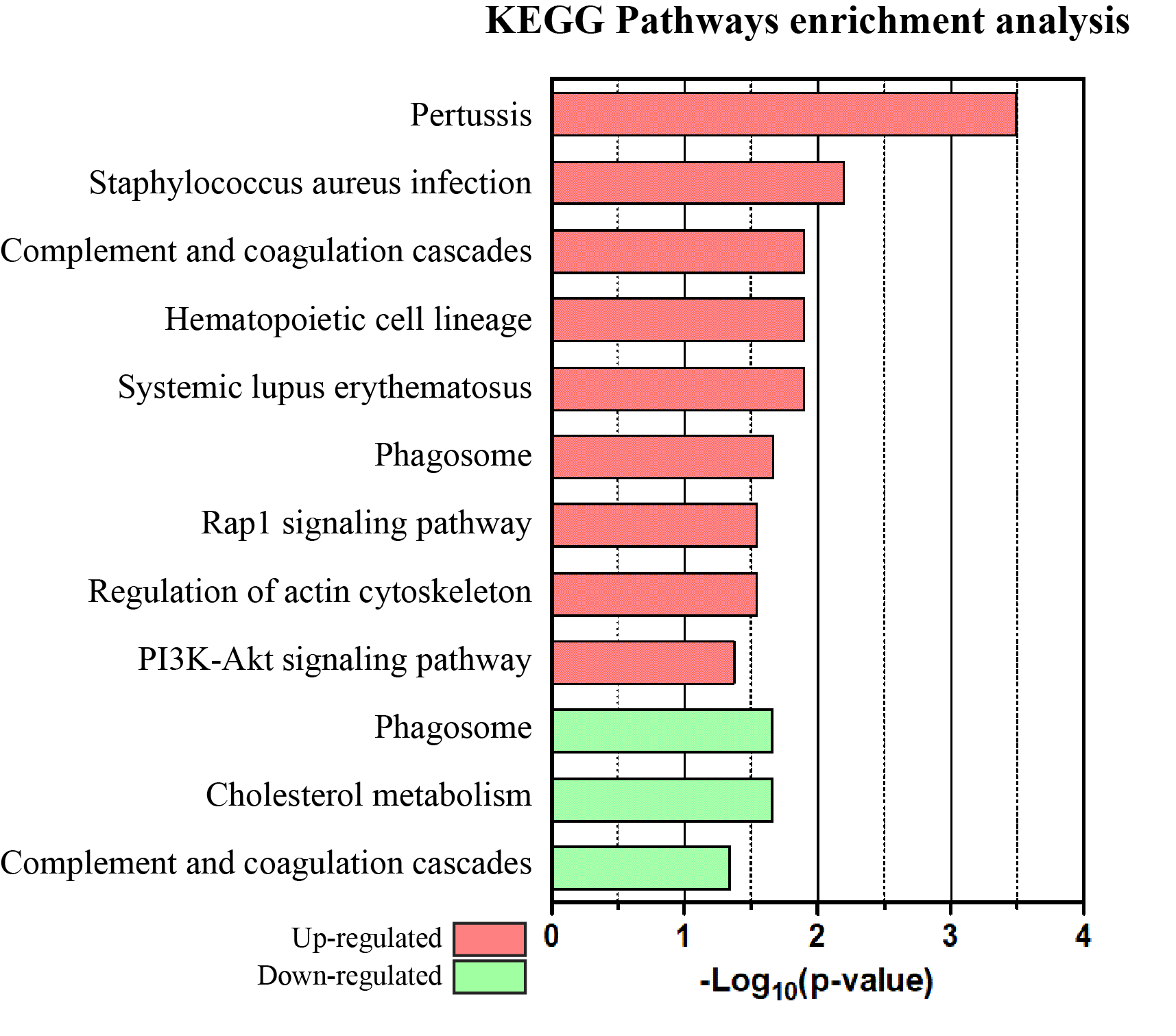

Supplement: Supplementary file 1 [file molecules-26-05992-s001.zip › Figure S3_Greco_2021.tif]

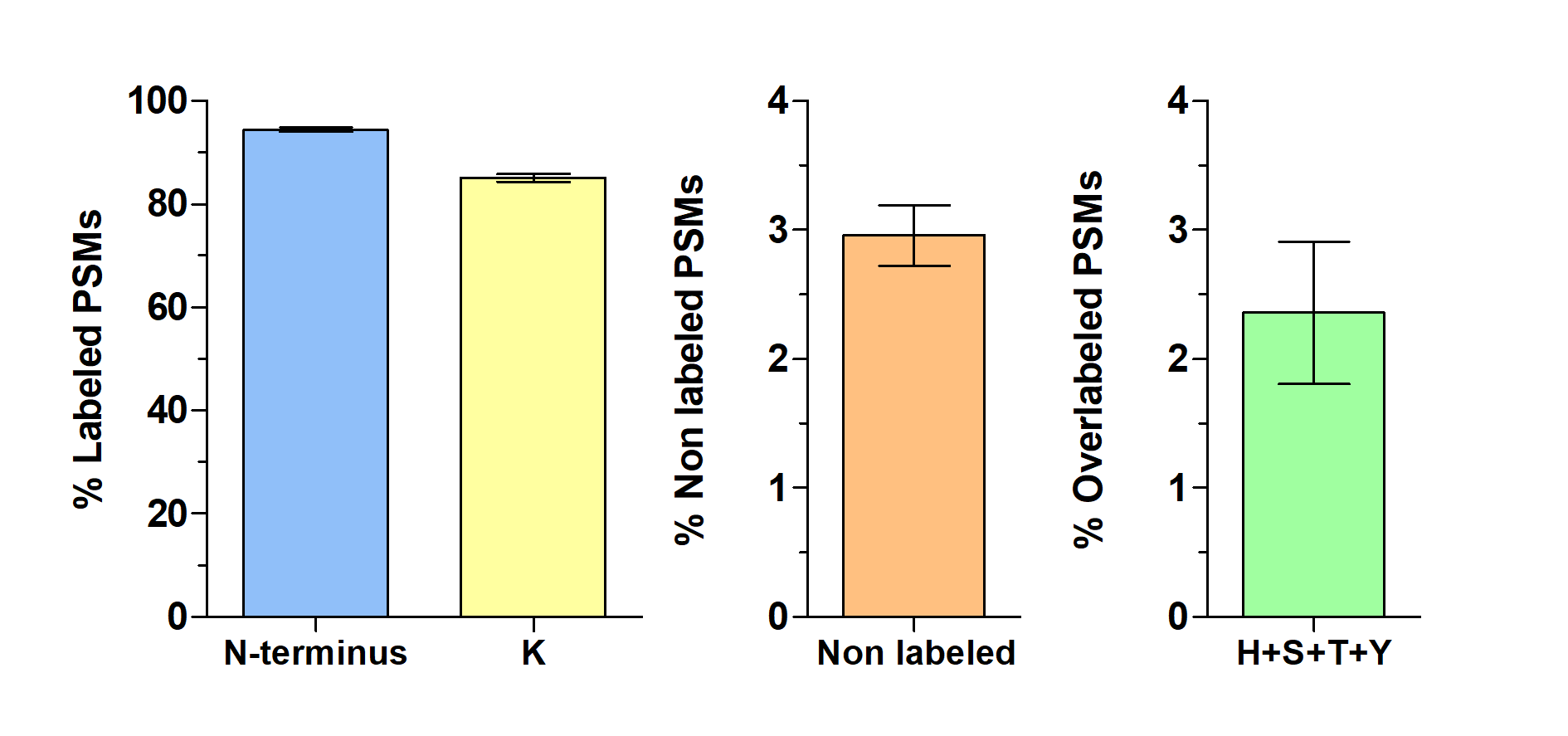

Supplement: Supplementary file 1 [file molecules-26-05992-s001.zip › Figure S5_Greco_2021.tif]
